# Timing and Scope of Genomic Expansion within Annelida: Evidence from Homeoboxes in the Genome of the Earthworm *Eisenia fetida*

**DOI:** 10.1093/gbe/evv243

**Published:** 2015-12-10

**Authors:** Allison S. Zwarycz, Carlos W. Nossa, Nicholas H. Putnam, Joseph F. Ryan

**Affiliations:** ^1^Whitney Laboratory for Marine Bioscience, University of Florida; ^2^Viterbo University; ^3^Department of Ecology and Evolutionary Biology, Rice University; ^4^Department of Biology, University of Florida

**Keywords:** earthworm genome, Hox, homeobox evolution, Annelida, *Eisenia fetida*

## Abstract

Annelida represents a large and morphologically diverse group of bilaterian organisms. The recently published polychaete and leech genome sequences revealed an equally dynamic range of diversity at the genomic level. The availability of more annelid genomes will allow for the identification of evolutionary genomic events that helped shape the annelid lineage and better understand the diversity within the group. We sequenced and assembled the genome of the common earthworm, *Eisenia fetida*. As a first pass at understanding the diversity within the group, we classified 363 earthworm homeoboxes and compared them with those of the leech *Helobdella robusta* and the polychaete *Capitella teleta*. We inferred many gene expansions occurring in the lineage connecting the most recent common ancestor (MRCA) of *Capitella* and *Eisenia* to the *Eisenia*/*Helobdella* MRCA. Likewise, the lineage leading from the *Eisenia*/*Helobdella* MRCA to the leech *H. robusta* has experienced substantial gains and losses. However, the lineage leading from *Eisenia*/*Helobdella* MRCA to *E. fetida* is characterized by extraordinary levels of homeobox gain. The evolutionary dynamics observed in the homeoboxes of these lineages are very likely to be generalizable to all genes. These genome expansions and losses have likely contributed to the remarkable biology exhibited in this group. These results provide a new perspective from which to understand the diversity within these lineages, show the utility of sub-draft genome assemblies for understanding genomic evolution, and provide a critical resource from which the biology of these animals can be studied.

## Background

The remarkable variation among animal body plans can be attributed to historical innovations in the genomic components underlying animal development. The evolutionary dynamics of the homeobox superfamily in particular, have played an important role in the evolution of animal form (Lewis 1978; McGinnis et al. 1984). Because of the well-known, highly conserved nature of the homeobox superfamily, we are able to distinguish genetic events that have taken place in the evolution of bilaterian species. Furthermore, analyzing the complete homeobox superfamily of a newly sequenced animal genome provides novel insight into the broader pattern of genomic evolution for that particular animal (Monteiro et al. 2006; Ryan et al. 2006, 2010; Holland et al. 2008; Martin and Holland 2014; Paps et al. 2015).

*Eisenia fetida* (also called red wigglers, compost worms, among other names) is a widespread non-burrowing earthworm that is known for its role in assessing terrestrial ecotoxicological levels (Spurgeon et al. 1994). These worms are particularly well known for their ability to compost rotting material and are important for waste management and environmental monitoring. Their ecological importance was highlighted in Charles Darwin’s final book, *The Formation of Vegetable Mould through the Action of Worms* (Darwin 1892). *Eisenia fetida* are highly amenable to laboratory manipulation and their success as a model system is clear from the more than 600 PubMed articles referencing *E. fetida*. Despite the utility of *E. fetida*, very few molecular resources exist, apart from a limited set of expressed sequence tag (EST) data (Pirooznia et al. 2007).

Like most other annelids, the basic body plan of *E. fetida* consists of a head followed by a segmented trunk and a tail. This simple body arrangement incorporates a tremendous amount of diversity including variation in number of segments, internal anatomy, as well as head and tail shapes. Homeobox transcription factors play a central patterning role during embryogenesis in most animals, and changes in the number, genomic arrangement, and regulation of these genes have been implicated in playing a major role in the diversification of animal body plans (Akam 1995). An important step in understanding the evolution of annelid body plan diversity is to understand the diversity of homeobox genes. There have been several studies of annelid homeobox genes (Dick and Buss 1994; Andreeva et al. 2001; Kulakova et al. 2007; Cho et al. 2012), but most of these have considered only the HOXL subclass of genes and have concentrated on a single annelid species.

To date, complete genome sequences are available from two annelids: the marine polychaete *C. teleta* and the freshwater leech *H. robusta* (Simakov et al. 2013). The *C. teleta* genome is highly conserved in terms of genomic architecture (e.g., macrosynteny, intron-retention, gene retention, and gene duplication) when compared with other bilaterian genomes, while the *H. robusta* genome is considered relatively dynamic (Simakov et al. 2013).

Notably, *E. fetida* shares a more recent common ancestor with *H. robusta* than either of them does with *C. teleta* (Purschke 2002; Erséus and Källersjö 2004; Weigert et al. 2014) making it a useful model for understanding the timing of the dynamic genomic events that have occurred in the lineage leading to the leech. The presence, absence, and arrangement of Hox genes are prime examples of the hyperdynamic nature of the *H. robusta* genome (Simakov et al. 2013). Understanding the timing and frequency of these changes along the lineages leading to the leech and earthworm will shed light on how this shift has influenced the evolution of these two animals.

To this end, we have sequenced and assembled a draft-quality genome of the earthworm *E. fetida*. Using this assembly, we are able to identify and phylogenetically classify the complete set of homeoboxes from *E. fetida*. Our analyses show that many of the gene duplication and loss events that are evident in the *H. robusta* genome predate the most recent common ancestor (MRCA) of *E. fetida* and*H. robusta*, and show that an extraordinary number of duplication events occurred in the earthworm lineage after it diverged from this ancestor.

## Materials and Methods

### Data Access

All genome sequencing data are available from the European Nucleotide Archive under the study accession: PRJEB10048 (http://www.ebi.ac.uk/ena/data/view/PRJEB10048, last accessed January 12, 2016). All alignments, trees, custom scripts, hidden Markov models (HMMs), and a detailed list of commands used in our analyses are available in our GitHub supplement: (https://github.com/josephryan/RyanLab/tree/master/2015-Zwarycz_et_al, last accessed January 12, 2016). The genome data can also be accessed through the *E. fetida* Genome Portal: http://ryanlab.whitney.ufl.edu/genomes/Efet/, last accessed January 12, 2016.

### Materials and Sequencing

Two adult, farm-raised *E. fetida* earthworms were crossed and produced 24 offspring. The digestive systems of the 26 worms were purged by being kept on moist paper clippings, out of dirt. The earthworms were washed with 70% ethanol prior to DNA extraction. DNA was extracted using Qiagen DNA Easy kit and Zymo Genomic DNA clean kit to further purify the DNA. Libraries were made with Nextera and sequenced on Illumina HiSeq2000 2 × 100PE. Sequencing was performed by the Beijing Genomics Institute (BGI).

### Error Correction and Adapter Trimming

Sequencing reads from each sample were concatenated and error correction was performed using the ErrorCorrectReads.pl program from Allpaths-LG version 44387 (Gnerre et al. 2011). Besides error correction, Allpaths-LG also estimates genome size based on k-mer spectrum. We used Cutadapt version 1.4.2 (Martin 2011) to remove adapter sequences from all error-corrected reads.

### Genome Assembly

After adapter trimming and error correction, we created a total of ten genome assemblies using the following assemblers: SOAPdenovo version 2.04 (Luo et al. 2012), ABySS version 3.81 (Simpson et al. 2009), and Platanus version 1.2.1 (Kajitani et al. 2014). Besides adjusting K-mer values, command-line parameters were mostly left as defaults.

### Assembly Evaluation

We evaluated each assembly using three primary criteria: 1) The number of *E. fetida* ESTs that aligned to an assembly, 2) the number of 248 highly conserved eukaryotic genes identified with CEGMA version 2.4 (Parra et al. 2007), and 3) the N50 statistic (table 1). We used BLAT version 35x1 (Kent 2002) to align 4,329 *E. fetida* ESTs available in GenBank (LIBEST_024375, LIBEST_026326, LIBEST_022256, and LIBEST_020813) and Isoblat version 0.3 (Ryan 2013) to gauge how well these ESTs mapped to each assembly.

**Table 1:** *Eisenia fetida* Assembly Statistics

Program (Parameters)	K-mer Size	N50 (bp)	Isoblat	CEGMA (Complete)	CEGMA (Partial)	Genome Length (Gb)
SOAPdenovo (defaults, max insert size = 300)	31	**1,852**	4,103/4,329 (95%)	**59**	**124**	1.05
	39	1,086	4,156/4,329 (96%)	53	110	1.28
	45	781	4,160/4,329 (96%)	46	105	1.47
	55	375	4,198/4,329 (97%)	39	91	2.07
	63	422	4,202/4,329 (97%)	36	93	2.13
ABySS (defaults)	31	61	4,237/4,329 (98%)	30	74	2.22
	45	94	4,237/4,329 (98%)	18	67	2.41
	63	165	**4,241/4,329 (98%)**	10	59	2.12
Platanus (defaults, m = 500)	32	414	4,108/4,329 (95%)	46	104	0.73
	45	307	4,119/4,329 (95%)	26	85	0.88

Note.—Bolded entries indicate best value in each column.

We aligned the most ESTs using our ABySS assembly with K = 63 (ABySS63 assembly), but this alignment produced the lowest CEGMA scores and had a very suboptimal N50. In contrast, we generated the highest CEGMA and N50 scores using our SOAPdenovo assembly with K = 31 (SOAP31 assembly). The difference in number of mapped ESTs between the ABySS63 assembly and our SOAP31 assembly was negligible, whereas the differences in CEGMA scores and N50 values between these two assemblies were substantial. In addition, the size of the SOAP31 assembly was much closer to the Allpaths-LG prediction than the size of the ABySS63 assembly. Based on these results we chose the SOAP31 assembly for all downstream analyses.

### Homeodomain Data Set and Alignment

We ran the hmmsearch program from HMMer version 3.1b1 (Eddy 2011) on a translated version of our final assembly. For this search, we used a custom homeobox HMM generated using hmmbuild on a FASTA file consisting of all homeodomains from HomeoDB (Zhong et al\\. 2008) that were 60 amino acids in length (hd60.hmm in GitHub supplement). The resulting search produced an alignment (to the HMM) that we converted from STOCKHOLM to FASTA (using http://sequenceconversion.bugaco.com, last accessed January 12, 2016). We then removed all regions that did not align to the HMM (i.e., insertions) using remove_gaps_from_hmmsearch_results.pl (GitHub supplement). We repeated this process on filtered protein models of *C. teleta, H. robusta*, and *Lottia gigantea* that were downloaded from the Joint Genome Institute web site and from *Crassostrea gigas*, which was downloaded from GigaDB (Fang et al. 2012). We labeled the sequences of *Cr. gigas* based on Paps et al. (2015).

For each species data set, we used BLASTP (version 2.2.31+) and two custom Perl scripts to identify sequences that were missed in our initial HMMsearch runs as has been recommended (Marlétaz et al. 2014). We first used our custom script hmmsearch_blast_combo.pl (GitHub supplement) to build a FASTA file with all of the sequences where a homeodomain was not recovered. We next ran a BLASTP with tabbed output and an e-value cutoff of 10 against all homeodomains from HomeoDB. We used our custom script parse_and_reblast_w_alignments.pl (GitHub supplement) to first identify hits with e-values below 0.01 (a more conservative 0.001 was used for *E. fetida*) and then to run a BLASTP search with default output on these searches. We extracted homeodomains from BLAST alignments and then aligned them to the complete set of amphioxous homeodomains available from HomeoDB with MAFFT (v7.158b). We removed any insertions outside of the canonical 60-amino acid homeobox and then appended the nonamphioxous homeodomains to our grand set. In total, we added 26 *C. teleta*, 31 *H. robusta*, 17 *L. gigantea*, and 191 *E. fetida* homeodomains. The large number of additional *E. fetida* homeoboxes is due mostly to the lack of available protein models for this species. All of the homeodomains, both from the primary and secondary searches (1,243 total), were included in our downstream analyses.

### Homeobox Phylogeny and Tree Generation

We used RAxML version 8.0.23 (Stamatakis 2006) to generate a maximum-likelihood (ML) tree from the aligned homeodomains of *E. fetida*, *C. teleta, H. robusta*, *L. gigantea*, and *Cr. gigas* (supplementary fig. S1, Supplementary Material online). We pruned taxa from this tree with terminal branches longer than 2.3 using the custom script branch_lengths_filter.pl (GitHub supplement), which removed poorly predicted or extremely divergent homeodomains. This cutoff removed 29 sequences, none of these were obvious homeodomains, but left three sequences (Ct_214198, Ct_199162, and Lg_132019) that appeared not to be homeodomains (sequences available in GitHub supplement). These were manually removed. This pruning left us with 1,209 homeodomains, including 466 *E. fetida*, 189 *Cr. gigas* (Mollusca), 155 *L. gigantea* (Mollusca), 271 *H. robusta* (Annelida), and 178 *C. teleta* (Annelida) sequences.

We separated the 1,209 sequences into classes, as designated by HomeoDB (Zhong et al\\. 2008) using the *Cr. gigas* class assignments as a guide. For each class-level data set, we generated an ML tree with RAxML, corresponding bootstraps with the autoMRE stopping criteria in RAxML, and a Bayesian tree using MrBayes version 3.2.3 (Ronquist and Huelsenbeck 2003). Alignments and details for phylogenetic runs are in the GitHub supplement. For the Bayesian trees, the potential Scale Reduction Factor (PSRF) values produced by “sump” for both the sum of all branch lengths (TL), and the shape parameters of the gamma distribution of rate variation (alpha) were very close to one (the largest difference was for the TL in the Other analysis: PSRF = 1.479514). According to the MrBayes manual, PSRF values close to 1.0 suggest a good sample from the posterior probability distribution.

### Homeobox Classification and Naming

For each class, we computed a majority rule consensus tree using RAxML (with the -J STRICT option) from the ML and Bayesian trees. We used these consensus trees to classify each homeodomain at the family level in accordance with the family assigned to the *Cr. gigas* homeodomain in Paps et al. (2015). We examined homeodomains where the consensus trees were inconclusive. In some cases, a homeodomain was excluded from a family clade in the consensus tree due to another homeodomain that was present in the clade in one of the trees but not the other (unstable taxa). In these cases we excluded the unstable taxa from the family, but classified the other homeodomains as family members. In cases where two or more partial homeodomains were identified on the same scaffold in the same direction and not completely overlapping, we collapsed these into a single homeodomain. When more than one homeodomain from a single species were assigned to the same family, we used the family name, followed by a numerical label from their definition number. If we were unable to assign a homeodomain to a family, it was given the class/subclass name followed by “HD” and a number >20 (e.g., HOXLHD23). All homeodomain assignments are available in the supplemental material, Supplementary Material online.

### Inferring Gene Duplications and Losses within Annelida

We used parsimony principles to infer the annelid evolutionary branch on which family-level gains and losses occurred. In this process, we estimated the ancestral condition based on the number of homeodomains present in a *Cr. gigas* family, unless the particular *Cr. gigas* family was 0, in which case the *L. gigantea* number was used (in most cases, these numbers were the same). We did not consider relationships within a family, as support for intrafamily relationships was mostly very low and would therefore require elaborate loss and gain scenarios. We classified each event (gains and losses) by determining the fewest number of events needed to explain the number of homeodomains identified in a particular family from the ancestral state. In cases where there were equally parsimonious explanations of the data, we chose the scenarios that maximized gains on external branches rather than internal branches. This biasing of events on terminal branches is justified based on the terminal branches of the three annelids all being over twice the length of the internal branch connecting the *Capitella*/Clitellata MRCA with the *Helobdella*/*Eisenia* MRCA (in fig. 1 of Weigert et al. 2014), suggesting that evolutionary events were more than twice as likely to occur on terminal branches.

**Fig. 1.—:**
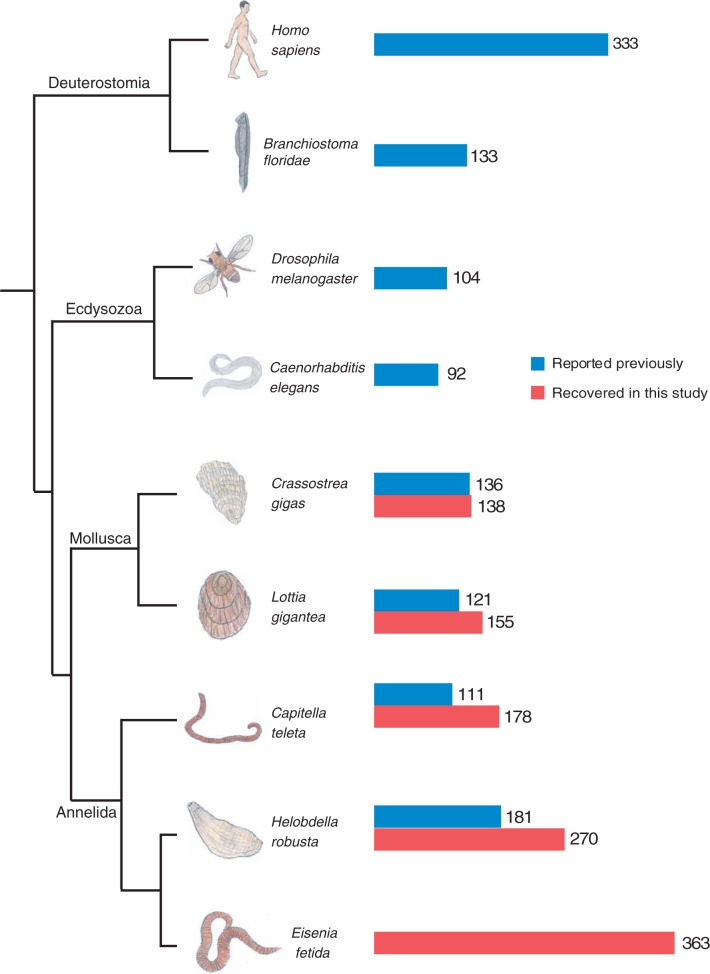
Phylogenetic relationship and total homeobox count of several bilaterian animals. The relationships of these taxa are based on multiple studies (Zhang et al. 2012; Simakov et al. 2013; Weigert et al. 2014). The bar graph to the right of taxa labels shows the number of homeoboxes reported previously in blue, and the number of homeoboxes we identified in our analyses in red. *Eisenia fetida* represents the highest reported total homeobox count among these animals.

### Identifying Partial E. fetida Homeoboxes

We used a custom Perl script filter_cterm_homeodomains.pl (GitHub supplement) to filter homeodomains that begin with 30 or more undetermined positions. Filtering these homeodomains prevented counting a single fragmented homeodomain twice.

### Counting Exact Pairs of Homeodomains

We used a custom Perl script find_duplicates.pl (GitHub supplement) to identify homeodomain pairs with the same exact amino acid sequences at nongap regions in each of the species in our analysis. We did not count sequences that begin with 30 or more undetermined positions, as these were not used in our final counts.

## Results

We sequenced and assembled the genome of the earthworm *E.fetida*. We identified 466 homeoboxes in this rough draft assembly. We collapsed 26 pairs of homeodomain fragments that were found on the same scaffold in the same direction and were monophyletic in our ML analysis (supplementary fig. S1, Supplementary Material online), which left 440 *E. fetida* homeodomains. Of the 440 sequences, 77 had 30 or more undetermined positions in the N-terminus. In the gene families of these C-terminal sequences we usually found corresponding N-terminal sequences. For this reason, we remove these C-terminal sequences from our final counts. These adjustments left us with a final count of 363 *E. fetida* homeodomains.

We retrieved 139 homeodomains from the oyster *Cr. gigas*, 155 from the limpet *L. gigantea*, 178 from the polychaete worm *C. teleta*, and 271 from the leech *H. robusta*. In all cases, we were able to identify additional homeoboxes from each annelid and molluscan genome using our approach of combining HMM and BLAST approach (fig. 1). It should also be noted that a similar approach was used for *Cr. gigas* (Paps et al. 2015), but that the others were discovered as part of whole-genome analyses, which were less targeted. We ran an extensive phylogenetic analysis using this comprehensive data set as a means to understand the nature of the *E. fetida* genome, and some of the evolutionary dynamics that led to this genome. In the process, we classified the *E. fetida*, *C. teleta*, *H. robusta*, and *L. gigantea* homeoboxes based on careful designations applied to *Cr. gigas* in a recent comprehensive analysis of the homeoboxes of this animal (Paps et al. 2015).

### Genome Assembly

We generated genomic reads from a mating pair of adult *E. fetida* and 24 offspring (315 million 100-bp paired-end reads on 300 bp inserts for a total of 630 million reads). We conducted a k-mer spectrum analysis with AllPaths-LG ErrorCorrectReads.pl, which provided a genome size estimate of 1,207,407,810 bases and a coverage estimate of 39×. After adapter trimming and error correction we generated ten assemblies of these combined reads using three different assembly algorithms and a range of k-mer values. Our best assembly was 1.05 Gb with an N50 of 1,850 bp (table 1).

### Class Designations

**Fig. 2.—:**
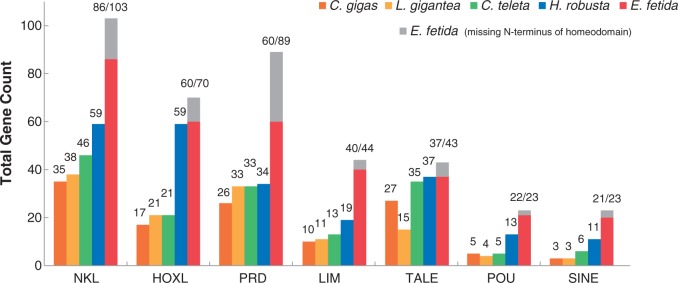
Total homeobox counts within classes/subclasses. Total homeobox counts for the largest homeobox classes (and subclasses) are shown for the mollusks *Cr. gigas* and *L. gigantea*, as well as the annelids *C. teleta*, *H. robusta*, and *E. fetida*. In all cases, *E. fetida* has the most homeoboxes, and *H. robusta* has the second-greatest number of homeoboxes relative all other animals in the comparison. Using a combination of searches with HMMs and BLAST, we added to the total homeoboxes that were found in previous studies. *Eisenia fetida* homeoboxes that are missing the first 30 amino acids of the homeodomain are counted as a gray bar. The bottom and top of the gray bar effectively provide an upper and lower bound of counts for that particular class of homeobox. The lower value is used throughout the text.

**Fig. 3.—:**
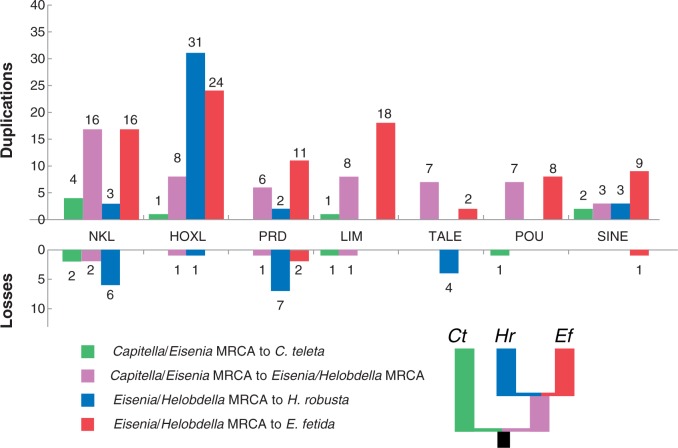
Inferred homeobox gains and losses in annelid lineages. Based on the distribution of homeobox genes on the phylogenetic tree in figure 1, we inferred gains and losses for four annelid lineages: 1) From the *Capitella*/*Eisenia* MRCA to *C. teleta*, 2) from the *Capitella*/*Eisenia* MRCA to the *Eisenia*/*Helobdella* MRCA, 3) from the *Eisenia*/*Helobdella* MRCA to *H. robusta*, and 4) from the *Eisenia*/*Helobdella* MRCA to *E. fetida*. The small tree next to the key shows lineage colors on the phylogeny.

We ran an ML analysis using the complete set of homeodomains from the five animals in our study to classify each into one of the major classes or subclasses using the *Cr. gigas* annotations as a guide. Figure 2 shows the distribution of homeoboxes according to class, with the most striking result being the large number of NKL, PRD, and LIM homeodomains in *E. fetida*. Similarly, we found a major expansion of HOXL homeoboxes in both *E. fetida* and *H. robusta* (fig. 3). To classify these genes at the family level, we conducted both ML and Bayesian phylogenetic analyses on each class with several smaller classes combined into a single group (other). We ran a strict consensus tree for each pair of Bayesian and ML analyses, and classified homeodomains at the family level if there was agreement between the two analyses. We were able to classify 263 out of the 363 *E. fetida* homeodomains (72%) at the family level using this consensus technique.

### ANTP Class—HOXL Subclass

**Fig. 4.—:**
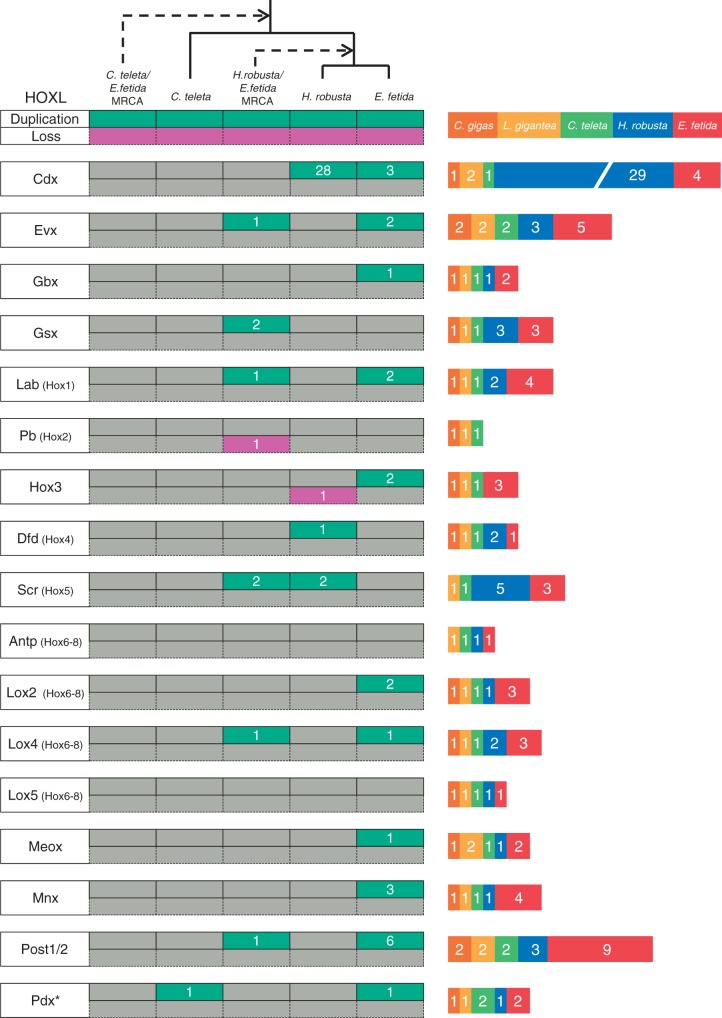
HOXL subclass (ANTP) evolutionary events and total homeobox count. Each green box on the left side of the figure represents one or more inferred duplication events, whereas each purple box represents one or more inferred losses. The number inside each green or purple box indicates the number of genes inferred to be gained or lost. An asterisk indicates more than one possible transition leading to the final homeobox count. The stacked bar graph represents the number of homeoboxes in each family for each species. Zero values do not appear in the graph. The dashed lines indicate ancestral nodes.

**Fig. 5.—:**
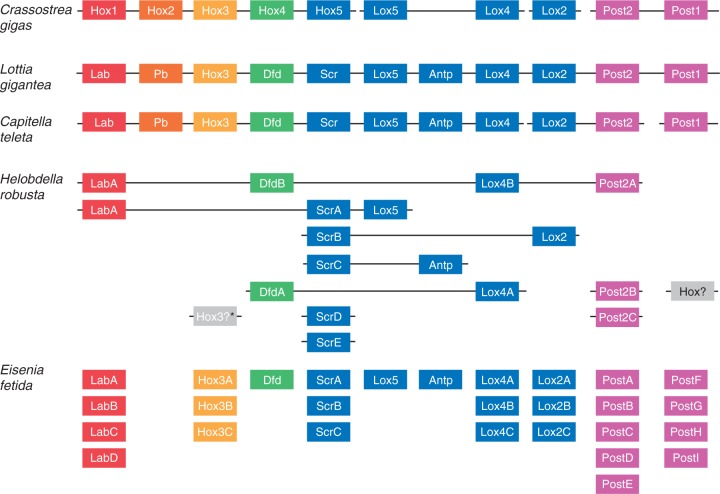
Hox clustering and count across two mollusks and three annelids. The *Cr. gigas* Hox cluster was obtained from Zhang et al. (2012). The *L. gigantea, H. robusta*, and *C. teleta* clusters were obtained from Simakov et al. (2013). *Crassostrea gigas*, *L. gigantea*, and *C. teleta* maintain a single copy of each Hox homeobox, whereas both *H. robusta* and *E. fetida* show multiple duplications in several families. The Post families experienced the largest number of duplications. However, we were unable to distinguish between the Post1 and Post2 in *E. fetida*. *Although a *H. robusta* Hox3 gene was identified in Simakov et al. (2013), we were unable to identify it in our analyses.

We identified 188 homeobox sequences belonging to the HOXL subclass of the ANTP class: 17 *Cr. gigas*, 21 *L. gigantea*, 21 *C. teleta*, 59 *H. robusta*, and 60 *E. fetida* (fig. 4; supplementary figs. S2–S4, Supplementary Material online). The expansion of the HOXL complement in *H. robusta* is due mostly due to a clade of 28 homeoboxes that form a larger clade with CDX homeoboxes (in both Bayes and ML trees). If this placement is true, it appears that the CDX gene duplicated 28 times in the lineage leading to *H. robusta* after diverging from the *E. fetida* lineage. This is an unprecedented degree of duplication in a Hox/ParaHox gene family. Besides the CDX duplications, we inferred eight duplication events in six HOXL families that occurred along the lineage leading to the *H. robusta*/*E. fetida* MRCA after the split from *C. teleta*. We also inferred 24 duplication events in 11 HOXL families in the *E. fetida* lineage after the split from *H. robusta* (figs. 4 and 5). We identify a loss of the Pb Hox gene in the lineage leading to the MRCA of *H. robusta* and *E. fetida*, and show that the Hox3 homeobox was lost in the lineage leading to *H. robusta* after diverging from *E. fetida*. Incidentally, Hox3 is reported to be present in *H. robusta* in Simakov et al. (2013), but we could not identify it in our analyses.

### ANTP Class—NKL Subclass

There was a major expansion of NKL homeoboxes (16 in 10 families) in the lineage leading to *E. fetida* after the *Eisenia/Helobdella* MRCA. We also inferred 16 gains of NKL homeoboxes in the lineage leading to the *Eisenia/Helobdella* MRCA and six losses in the lineage leading to *H. robusta* from this ancestor*.* Finally, we inferred the loss of four NKL-class homeoboxes in the lineage leading to the Capitellidae/Clitellata ancestor, which includes the Vax, Nk4, Msx, and Hlx families (supplementary figs. S5–S8, Supplementary Material online).

### PRD Class

As in the ANTP class, we deduced in the PRD class an extraordinary number of homeobox gene duplications (11 in seven families) to have occurred in the lineage leading to *E. fetida* after diverging from the *Eisenia/Helobdella* MRCA. In addition, we inferred six gains in the *Eisenia/Helobdella* ancestor in six different families (supplementary figs. S9–S12, Supplementary Material online). We inferred seven losses in the lineage leading to *H. robusta* after the *Eisenia/Helobdella* MRCA and also the loss of the Pax4/6 family in the lineage leading to the Capitellidae/Clitellata ancestor. We also identified an additional Pax6 homeodomain in *C. teleta* (Ct_Pax6A) where there were thought to be only one (Seaver et al. 2012).

### LIM Class

We inferred that the number of LIM class homeobox genes more than doubled in the lineage leading to *E. fetida* after the *Eisenia/Helobdella* MRCA (18 duplications occurring in six of the nine families). There were also six duplications in the Islet family that occurred in the lineage leading to the *Eisenia/Helobdella* MRCA after the split from *Capitella* (supplementary figs. S13–S16, Supplementary Material online).

### Other Classes

We have highlighted the largest homeobox classes, all of which experienced major expansions in the *E. fetida* lineage. Most of the other classes also experienced expansions in the *E. fetida* lineage, especially the SINE class where we inferred nine duplications in the lineage leading to *E. fetida* after the *Eisenia/Helobdella* MRCA (supplementary figs. S17–S32, Supplementary Material online).

### Effects of Heterozygosity

In the present study, we sequenced two farm-raised *E. fetida* and their offspring, which almost certainly will produce a highly heterozygous sequencing pool. At high levels of heterozygosity, a genome assembly can encompass multiple haplotypes and lead to genomic elements, including homeodomains, being represented multiple times. This potentially could have a major effect on our findings. Homeoboxes are under extraordinary high levels of negative selection, and it is highly unlikely that there would be variation between haplotypes in the amino acid sequences of homeodomains. To test whether our counts of *E. fetida* homeodomains were inflated due to heterozygosity, we counted the number of homeodomain pairs with the same exact amino acid sequences at nongap regions and compared that number to the other species in our analyses (table 2). The 19 pairs of matching homeodomains we identified in *E. fetida* were only four greater than the 15 that we found in each of the other annelid genomes. If these four were due to heterozygosity, it would not effect our conclusions.

**Table 2:** Counts of Homeodomain Pairs with the Exact Amino Acid Sequences at Nongap Regions

Species	*Eisenia fetida*	*Capitella teleta*	*Helobdella robusta*	*Lottia gigantea*	*Crassostrea gigas*
Duplicates	19	15	15	13	2

## Discussion

We have sequenced and assembled the genome of the earthworm *E. fetida*. We have also annotated 363 *E. fetida* homeoboxes in an effort to understand the evolutionary dynamics that shaped its genome. We have inferred the timing of an extraordinary number of evolutionary genomic events in the form of homeobox gene duplications and losses that have occurred in the lineage leading to *E. fetida*.

### Rates of Gene Gain and Gene Loss

Consistent with previous studies (Simakov et al. 2013), we find that numerous homeobox gains and losses occurred in the lineage leading from the Capitellidae/Clitellata ancestor to the lineage leading to *H. robusta*. Using data from the earthworm genome, we show that many of these events occurred prior to the MRCA of *H. robusta* and *E. fetida*, and that although the *H. robusta* lineage continued to experience additional homeobox gains and losses after the split from this ancestor, the gene duplication process was accelerated in the lineage leading to *E. fetida*. Based on the pattern that we observe in the homeobox superfamily of genes, we propose that dynamics of genomic gain and loss rates is generalizable across each of these annelid lineages.

### *Eisenia fetida* Hox Genes

We recovered 28 Hox genes from the *E. fetida* genome (fig. 5). It will be interesting to see the extent of clustering in the *E. fetida* Hox genes once there is higher genomic resolution. This resolution will provide some insight into the nature of gene expansion (i.e., individual duplications vs. large segmental duplications) in these lineages. For example, if these 28 Hox genes are situated in multiple Hox clusters, it would suggest that gene expansion in the *E. fetida* lineage was due to large segmental duplications and possibly whole-genome duplication(s).

### Utility of Subdraft-Level Genomes

There are challenges and caveats associated with these subdraft-level genomes. For example, draft genome assemblies, with N50s considerably higher than the assembly reported here, have been shown to contain many errors (Han et al. 2013). These errors can be in the form of gene-number inflation (Alkan et al. 2011) and missing coding exons (Denton et al. 2014). A major cause of annotation error comes from incomplete assembly, which leads to genes fragmented across multiple scaffolds, but also can be due to local instances of misassembly as well as local sequence variation. Much of the gene-number inflation comes from counting a gene encompassed on multiple scaffolds as multiple genes (Alkan et al. 2011). However, in the current study, we are searching for a stretch of DNA that is usually no more than 180 nucleotides and over 90% of the genome is assembled in chunks bigger than 180 nucleotides. We do find that introns can lead to homeoboxes being fragmented across multiple scaffolds, but by not counting homeodomains that are missing 50% of the N-terminus we are able to generate conservative counts. It is possible that we have missing coding exons in our assembly, but these are likely to contain zero or only a small number of *E. fetida* homeoboxes, and if present in our assembly would only enforce our findings.

By using a subdraft level genome, we were able to identify an unprecedented number of homeoboxes in an invertebrate animal and in the process, better understand how annelid genomes have evolved. Assembling large (>1 Gb) genome sequences to a high-quality level requires the use of multiple technologies (e.g., Illumina paired-end sequencing + Fosmids, BACs, PacBio, mate pairs, etc.) and a large team and is expensive and time consuming. We were able to generate a subdraft level genome assembly using only Illumina technology and a small team of researchers in a reasonable amount of time. It is clear from these analyses that genome sequences of this quality can be useful for understanding broad principles in genome evolution, and we have shown that this approach is able to provide a better understanding of animal genome dynamics within a localized clade of animals. By scaling this approach, it is possible to gain a broad evolutionary perspective of how genomes have evolved across animals, and possibly reveal general evolutionary principles.

### Implications of Gene Duplications on E. fetida Embryogenesis

Expanding taxon sampling will be critical for determining more precise timing for the described genomic expansion events. Genome sequencing of additional ingroup taxa (e.g., annelids from within Terebelliformia, Arenicolidae, Opheliidae, and Echiura) is a necessary step toward better resolution of the timing of events. This will in turn be critical for correlating events with the origin of synapomorphies in various clades of Sedentaria (a clade of annelids that include *Capitella*, *Helobdella*, and *Eisenia*) (Weigert et al. 2014).

Annelids (and many other spiralians) have a highly stereotyped cleavage program called spiral cleavage (Shankland and Seaver 2000). Earthworms have a spiral cleavage program that is a significant departure from other annelids (Anderson 2013). Homeobox genes are critical throughout embryogenesis including early development (Carroll et al. 2013). This huge expansion in these important developmental genes represents a prime target for research into understanding the changes in early earthworm development. The *E. fetida* genome sequence provides an indispensible tool from which the genetic causes of this developmental diversity can be investigated.

## Supplementary Material

Supplementary figures S1–S32 are available at *Genome Biology and Evolution* online (http://www.gbe.oxfordjournals.org/).

Supplementary Data
