# Supplementary material for: Timing and Scope of Genomic Expansion within Annelida: Evidence from Homeoboxes in the Genome of the Earthworm Eisenia fetida
Source: Genome Biol Evol. 2015 Dec 10;8(1):271–81. doi: 10.1093/gbe/evv243 (PMC4758240; doi:10.1093/gbe/evv243)
Supplement: Supplementary Data [file supp_8_1_271__index.html]

Timing and scope of genomic expansion within Annelida: evidence from homeoboxes in the genome of the earthworm Eisenia fetida — Timing and Scope of Genomic Expansion within Annelida: Evidence from Homeoboxes in the Genome of the Earthworm Eisenia fetida — Supplementary Data 

# Timing and Scope of Genomic Expansion within Annelida: Evidence from Homeoboxes in the Genome of the Earthworm *Eisenia fetida*

## Supplementary Data

files

- Supplementary Data - pdf file
