# Supplementary material for: Timing and Scope of Genomic Expansion within Annelida: Evidence from Homeoboxes in the Genome of the Earthworm Eisenia fetida
Source: Genome Biol Evol. 2015 Dec 10;8(1):271–81. doi: 10.1093/gbe/evv243 (PMC4758240; doi:10.1093/gbe/evv243)

Supplemental Fig. S1

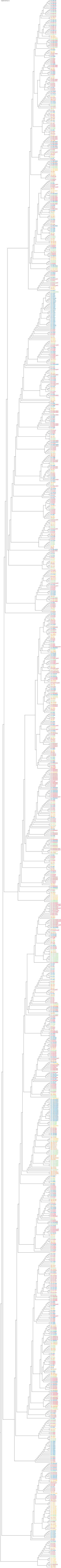

Supplemental Fig. S2

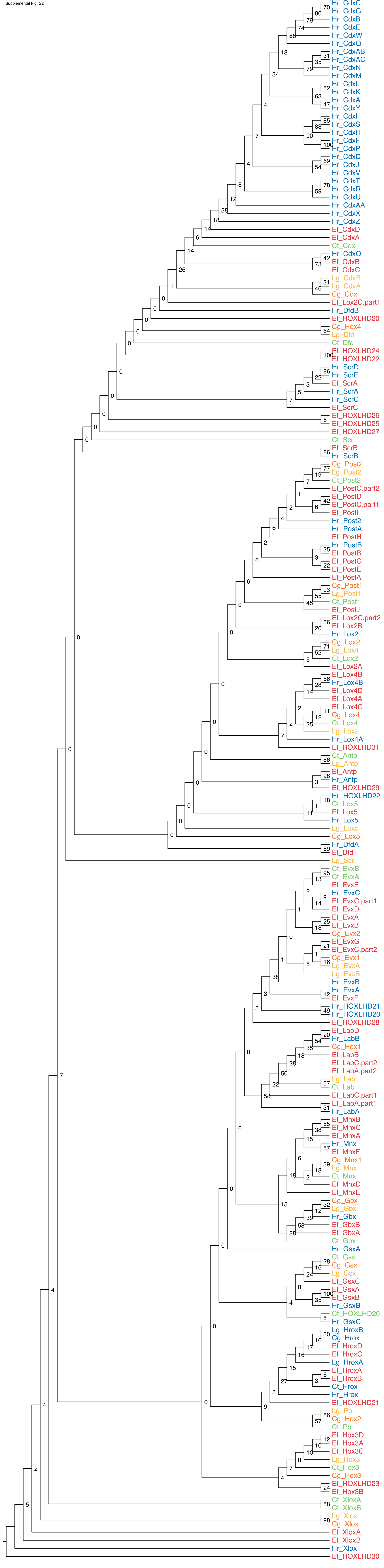

Supplemental Fig. S3

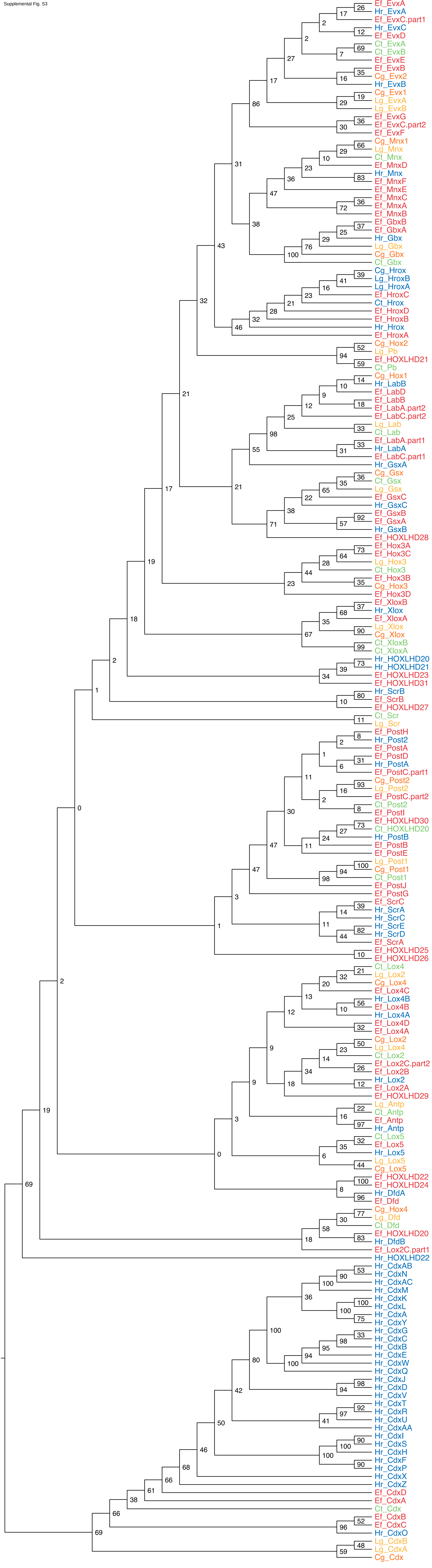

Supplemental Fig. S4

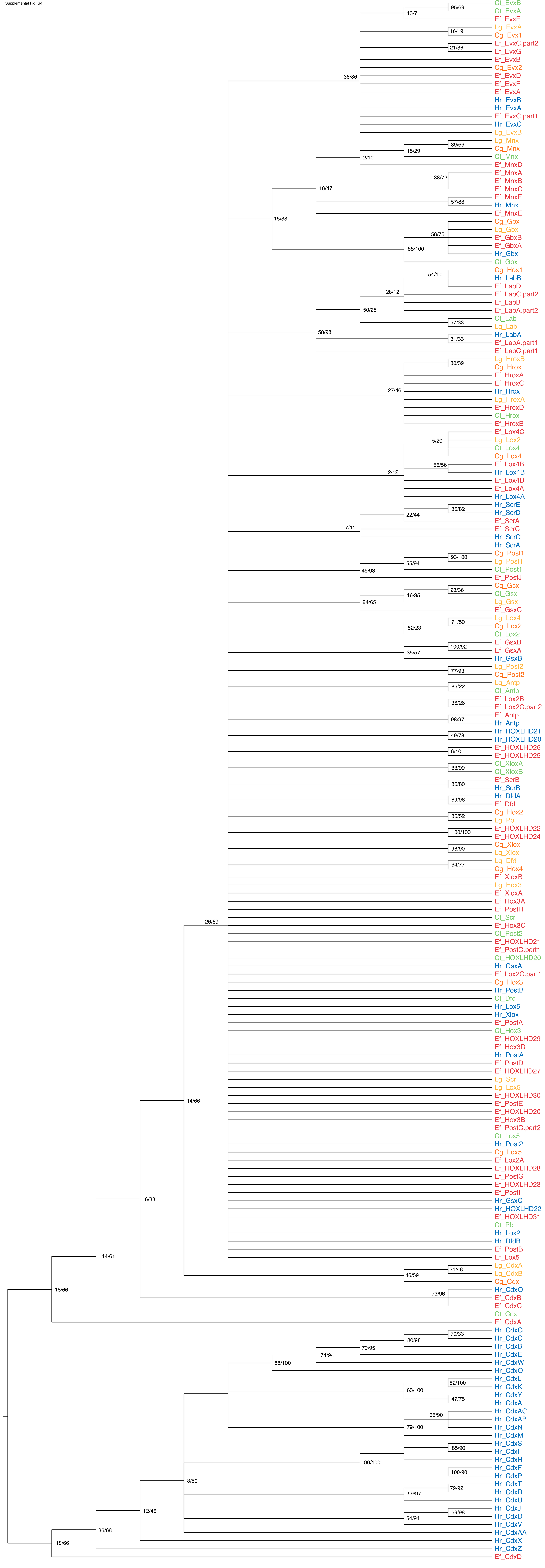

Supplemental Fig. S5

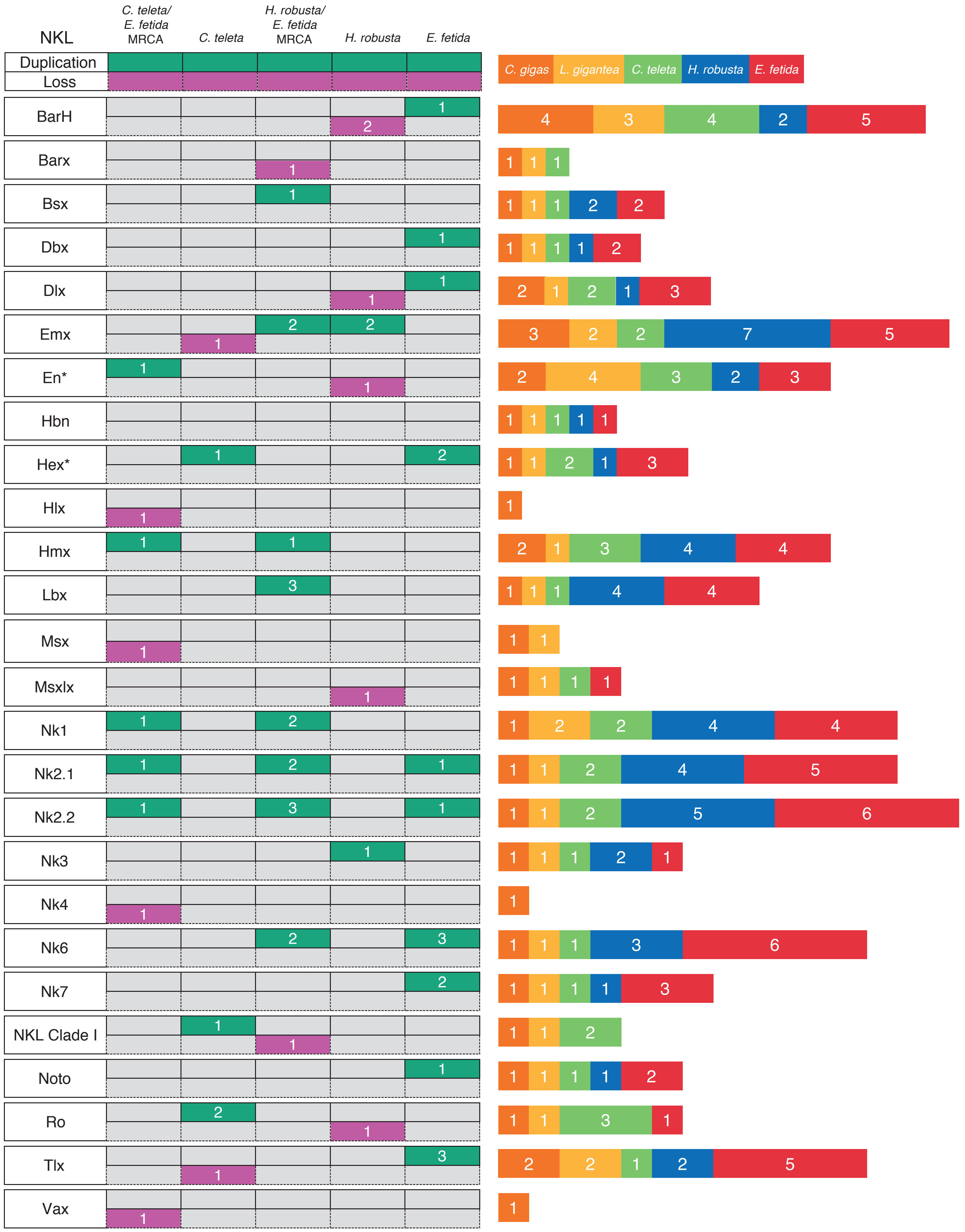

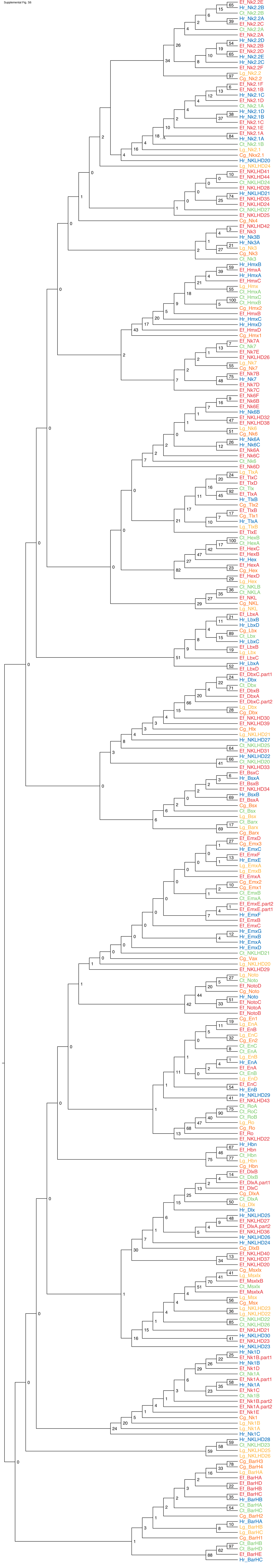
Ef_Nk2.2E
Hr_Nk2.2B
Ct_Nk2.2B
Hr_Nk2.2A
Ef_Nk2.2C
Ct_Nk2.2A
Ef_Nk2.2A
Hr_Nk2.2D
Ef_Nk2.2B
Ef_Nk2.2D
Hr_Nk2.2E
Hr_Nk2.2C
Ef_Nk2.2F
Lg_Nk2.2
Cg_Nk2.2
Ef_Nk2.1F
Ef_Nk2.1B
Hr_Nk2.1C
Ef_Nk2.1D
Ct_Nk2.1A
Hr_Nk2.1D
Hr_Nk2.1B
Ef_Nk2.1C
Ef_Nk2.1E
Ef_Nk2.1A
Hr_Nk2.1A
Ct_Nk2.1B
Lg_Nk2.1
Cg_Nkx2.1
Hr_NKLHD20
Lg_NKLHD24
Ef_NKLHD41
Ef_NKLHD44
Ct_NKLHD24
Hr_NKLHD28
Hr_NKLHD21
Ef_NKLHD35
Ef_NKLHD24
Ct_NKLHD27
Ef_NKLHD25
Cg_Nk4
Ef_NKLHD42
Ef_Nk3
Hr_Nk3B
Hr_Nk3A
Lg_Nk3
Cg_Nk3
Ct_Nk3
Hr_HmxB
Ef_HmxA
Hr_HmxA
Ef_HmxC
Lg_Hmx
Ct_HmxA
Ct_HmxC
Ct_HmxB
Cg_Hmx2
Ef_HmxB
Hr_HmxC
Hr_HmxD
Ef_HmxD
Cg_Hmx1
Ef_Nk7A
Ct_Nk7
Ef_Nk7E
Ef_NKLHD26
Lg_Nk7
Cg_Nk7
Ef_Nk7B
Hr_Nk7
Ef_Nk7D
Ef_Nk7C
Ef_Nk6F
Ef_Nk6B
Ef_Nk6E
Hr_Nk6B
Ef_NKLHD32
Ef_NKLHD38
Lg_Nk6
Cg_Nk6
Hr_Nk6A
Hr_Nk6C
Ef_Nk6A
Ef_Nk6C
Ct_Nk6
Ef_Nk6D
Lg_TlxA
Ef_TlxC
Ef_TlxD
Ct_Tlx
Ef_TlxA
Hr_TlxB
Cg_Tlx2
Ef_TlxB
Cg_Tlx1
Hr_TlxA
Lg_TlxB
Ef_TlxE
Ct_HexB
Ct_HexA
Ef_HexC
Ef_HexB
Hr_Hex
Ef_HexA
Cg_Hex
Ef_HexD
Lg_Hex
Ct_NKLB
Ct_NKLA
Ef_NKL
Cg_NKL
Lg_NKL
Ef_LbxA
Hr_LbxB
Hr_LbxD
Cg_Lbx
Ct_Lbx
Hr_LbxC
Ef_LbxB
Lg_Lbx
Ef_LbxC
Hr_LbxA
Ef_LbxD
Ef_DbxC.part1
Hr_Dbx
Ct_Dbx
Ef_DbxB
Ef_DbxA
Ef_DbxC.part2
Lg_Dbx
Cg_Dbx
Ef_NKLHD30
Ef_NKLHD39
Cg_Hlx
Lg_NKLHD21
Hr_NKLHD27
Ct_NKLHD25
Ef_NKLHD31
Hr_NKLHD22
Ct_NKLHD20
Ef_NKLHD33
Ef_BsxC
Hr_BsxA
Ef_BsxB
Ef_NKLHD34
Hr_BsxB
Ef_BsxA
Cg_Bsx
Ct_Bsx
Lg_Bsx
Ct_Barx
Lg_Barx
Cg_Barx
Ef_EmxD
Cg_Emx3
Hr_EmxC
Ef_EmxF
Hr_EmxE
Lg_EmxA
Lg_EmxB
Ef_EmxA
Cg_Emx2
Cg_Emx1
Ct_EmxB
Ct_EmxA
Ef_EmxE.part2
Ef_EmxE.part1
Hr_EmxF
Ef_EmxB
Ef_EmxC
Hr_EmxG
Hr_EmxB
Hr_EmxA
Hr_EmxD
Ct_NKLHD21
Cg_Vax
Lg_NKLHD20
Ef_NKLHD29
Lg_Noto
Ct_Noto
Ef_NotoD
Cg_Noto
Hr_Noto
Ef_NotoC
Ef_NotoA
Ef_NotoB
Cg_En1
Lg_EnA
Ef_EnB
Lg_EnC
Cg_En2
Ct_EnC
Ct_EnA
Lg_EnB
Hr_EnA
Ef_EnA
Ct_EnB
Lg_EnD
Ef_EnC
Hr_EnB
Hr_NKLHD29
Ef_NKLHD43
Ct_RoA
Ct_RoC
Ct_RoB
Lg_Ro
Cg_Ro
Ef_Ro
Ef_NKLHD22
Hr_Hbn
Ef_Hbn
Ct_Hbn
Lg_Hbn
Cg_Hbn
Ef_DlxB
Ct_DlxB
Ef_DlxA.part1
Ef_DlxC
Cg_DlxA
Ct_DlxA
Lg_Dlx
Hr_Dlx
Hr_NKLHD25
Ef_NKLHD27
Ef_DlxA.part2
Ef_NKLHD36
Hr_NKLHD26
Hr_NKLHD24
Cg_DlxB
Ef_NKLHD40
Ef_NKLHD37
Ef_NKLHD20
Cg_Msxlx
Lg_Msxlx
Ef_MsxlxB
Ct_Msxlx
Ef_MsxlxA
Lg_Msx
Cg_Msx
Lg_NKLHD23
Lg_NKLHD22
Ct_NKLHD22
Ct_NKLHD26
Ef_NKLHD21
Hr_NKLHD30
Ef_NKLHD23
Hr_NKLHD23
Hr_Nk1D
Ef_Nk1B.part1
Hr_Nk1B
Ef_Nk1D
Ct_Nk1A
Ef_Nk1A.part1
Hr_Nk1A
Ef_Nk1C
Ct_Nk1B
Ef_Nk1B.part2
Ef_Nk1A.part2
Ef_Nk1E
Cg_Nk1
Lg_Nk1B
Lg_Nk1A
Hr_Nk1C
Hr_NKLHD28
Ct_NKLHD23
Lg_NKLHD25
Lg_NKLHD26
Cg_BarH3
Cg_BarH4
Lg_BarHA
Ef_BarHA
Ef_BarHD
Ef_BarHB
Ef_BarHC
Hr_BarHB
Ct_BarHA
Ct_BarHC
Cg_BarH2
Hr_BarHA
Lg_BarHB
Lg_BarHC
Cg_BarH1
Ct_BarHB
Ct_BarHD
Ef_BarHE
Hr_BarHC

Supplemental Fig. S7

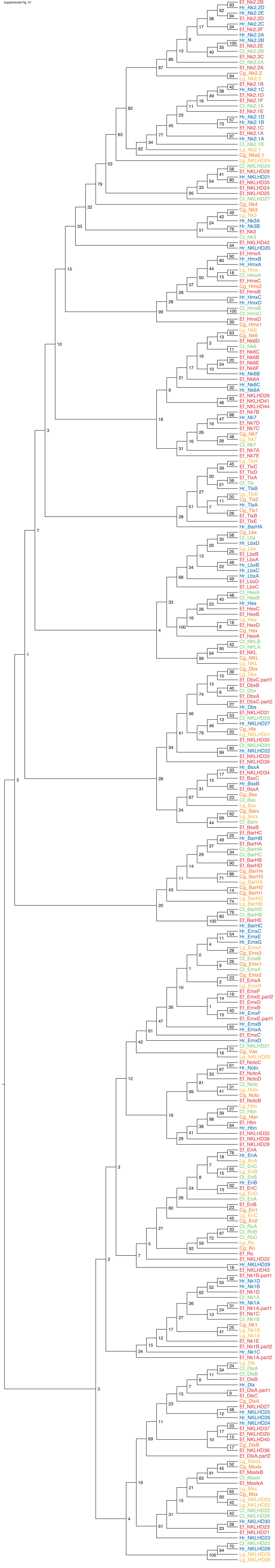

Supplemental Fig. S8

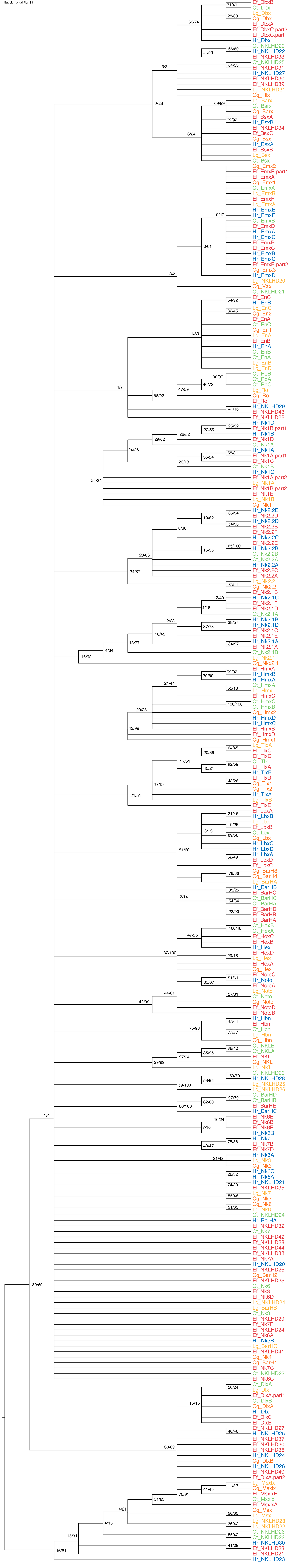

Supplemental Fig. S9

PRD		*C. teleta/ E. fetida* MRCA	*C. teleta*	*H. robusta/ E. fetida* MRCA	*H. robusta*	*E. fetida*
Arx	Duplication				1	
	Loss					1
Dmbx	Duplication					
	Loss					
Drgx	Duplication					
	Loss					1
Gsc	Duplication					
	Loss				2	
Otp	Duplication	1		1		
	Loss					
Otx	Duplication			1	1	
	Loss					
Pax3/7	Duplication			1		
	Loss					
Pax4/ 6	Duplication					
	Loss	1				
Pax6	Duplication	1				1
	Loss					
Pitx	Duplication					3
	Loss					
Prop	Duplication			1		
	Loss					
Prxx	Duplication			1		1
	Loss					
Rax	Duplication					1
	Loss					
Repo	Duplication					
	Loss				1	
Shox	Duplication			1		2
	Loss					
Uncx*	Duplication	1				1
	Loss				1	
Vsx*	Duplication	1				2
	Loss				1	
PRD1	Duplication					
	Loss			1		
PRD6	Duplication					
	Loss				1	
PRD7	Duplication					
	Loss					
PRD8	Duplication					
	Loss				1	

PRD	*C. gigas*	*L. gigantea*	*C. teleta*	*H. robusta*	*E. fetida*
Arx	1	1	1	2	
Dmbx	1	1	1	1	1
Drgx	1	1	1	1	
Gsc	2	1	2		2
Otp	1	1	2	3	3
Otx	1	1	1	3	2
Pax3/7	1	1	1	2	2
Pax4/ 6	1	1			
Pax6	1	1	2	2	3
Pitx	1	1	1	1	4
Prop	1	1	1	2	2
Prxx	1	1	1	2	3
Rax	1	1	1	1	2
Repo	1	1	1		1
Shox	1	1	1	2	3
Uncx*	1	1	2	1	3
Vsx*	2	3	3	2	5
PRD1	1	1	1		
PRD6	1		1		1
PRD7	1	1	1	1	1
PRD8	1	1	1		1

Supplemental Fig. S10

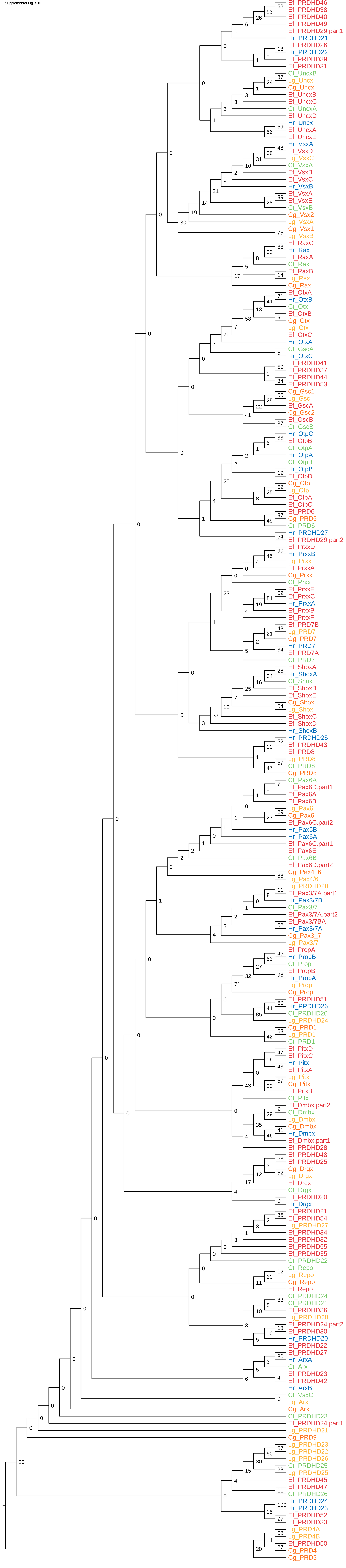

Supplemental Fig. S11

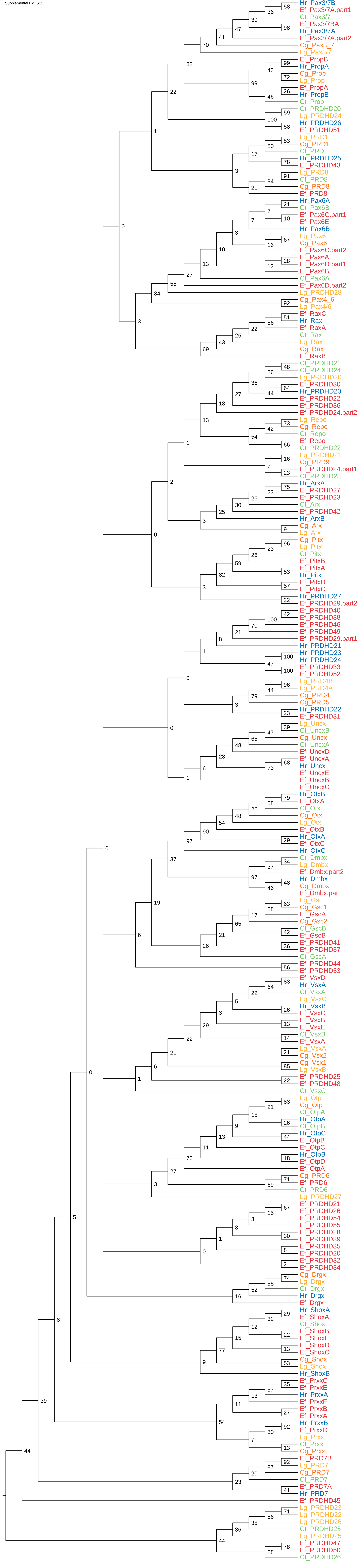

Supplemental Fig. S12

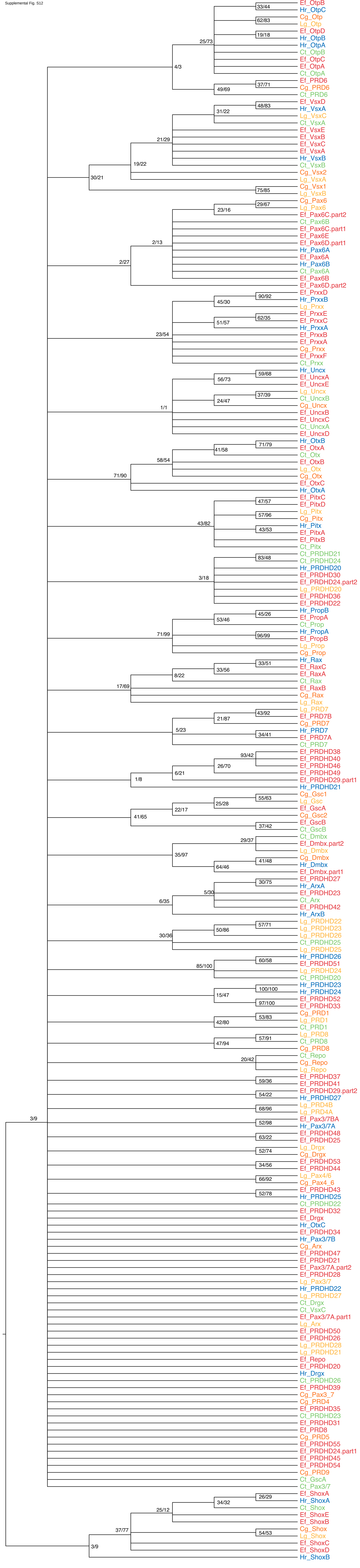

Supplemental Fig. S13

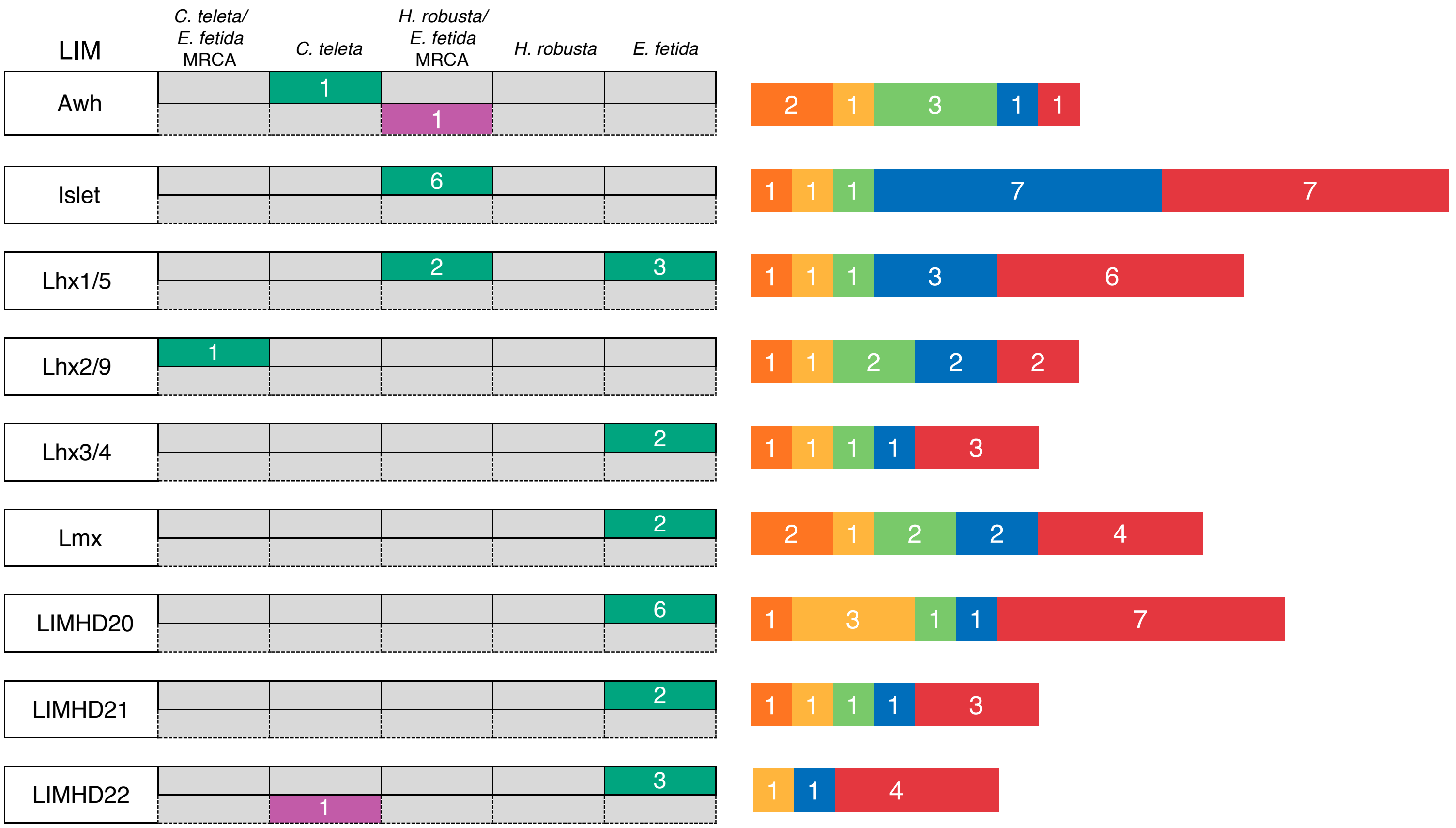

Supplemental Fig. S14

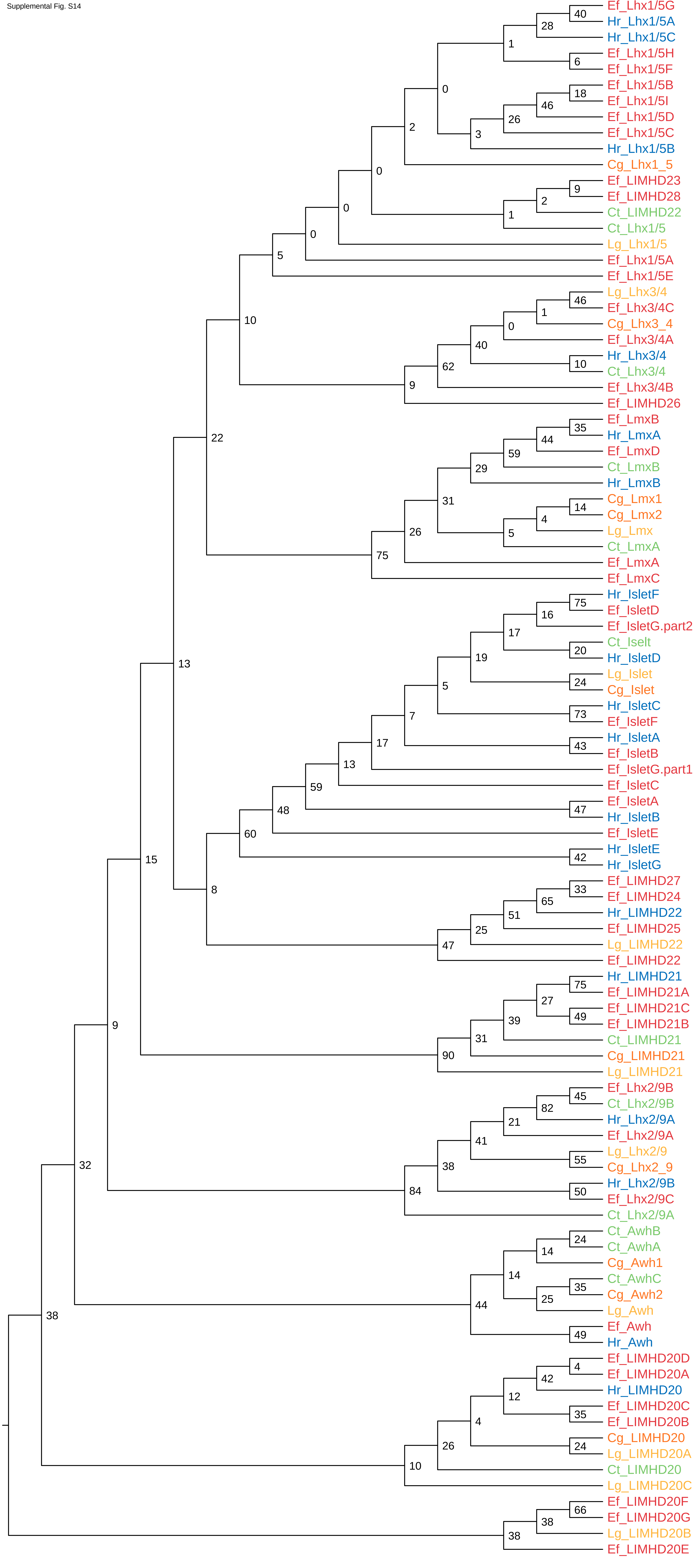

Supplemental Fig. S15

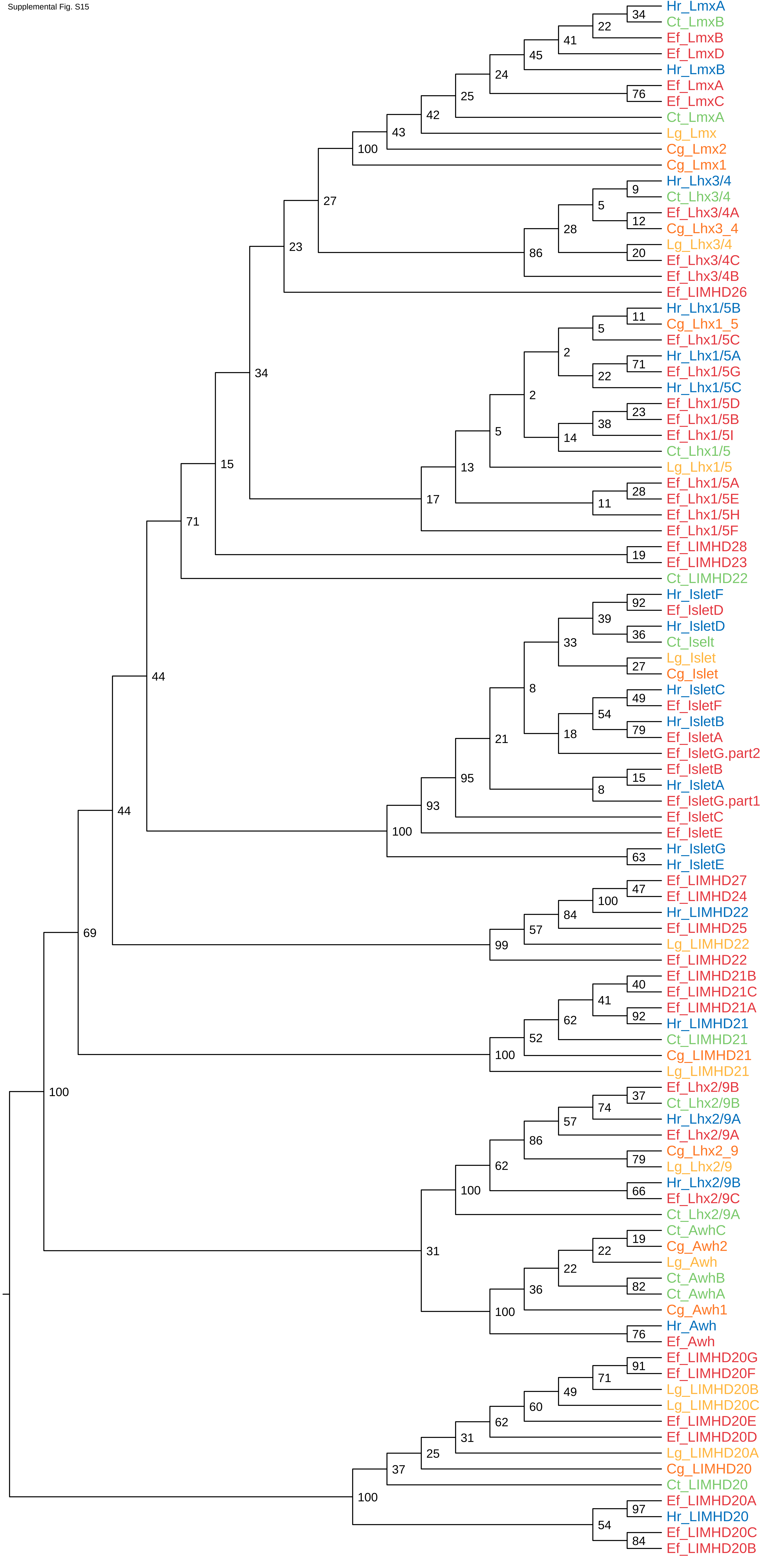

Supplemental Fig. S16

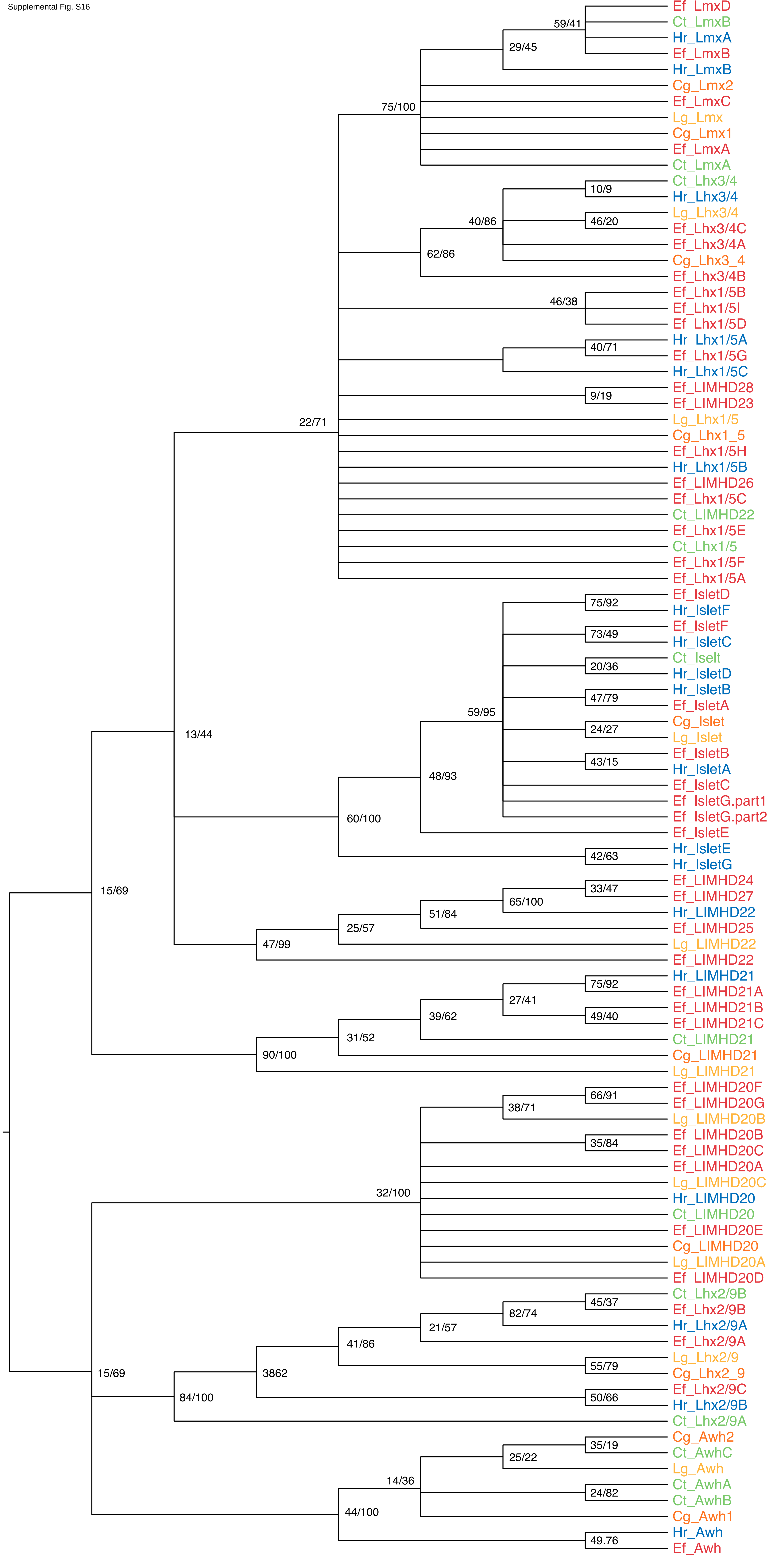

Supplemental Fig. S17

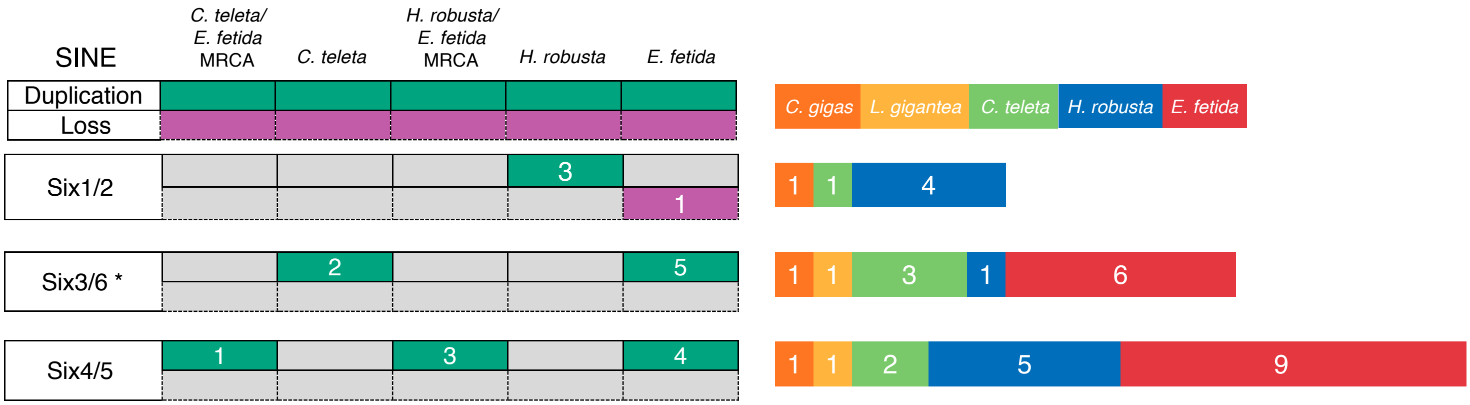

Supplemental Fig. S18

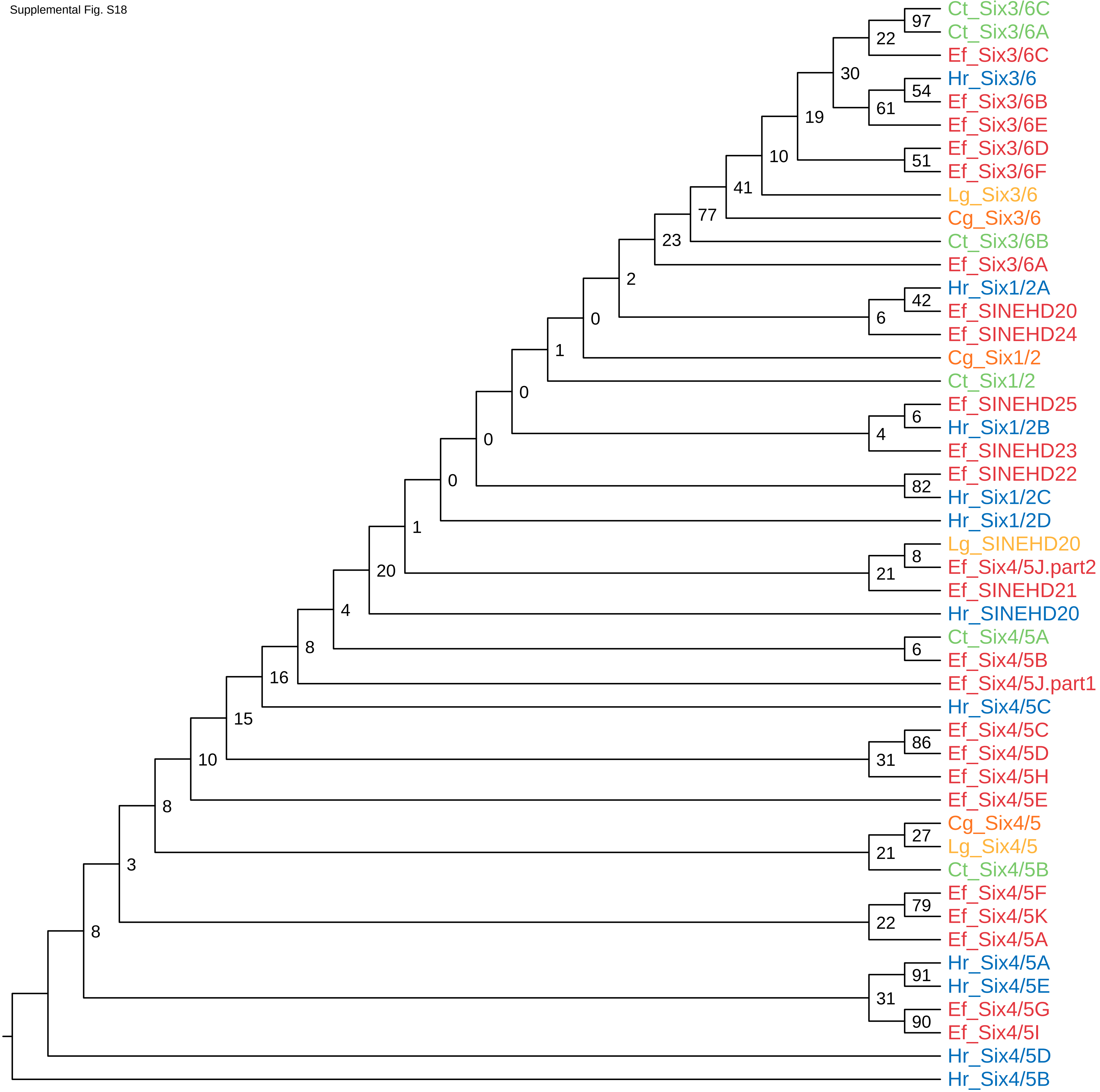

Supplemental Fig. S19

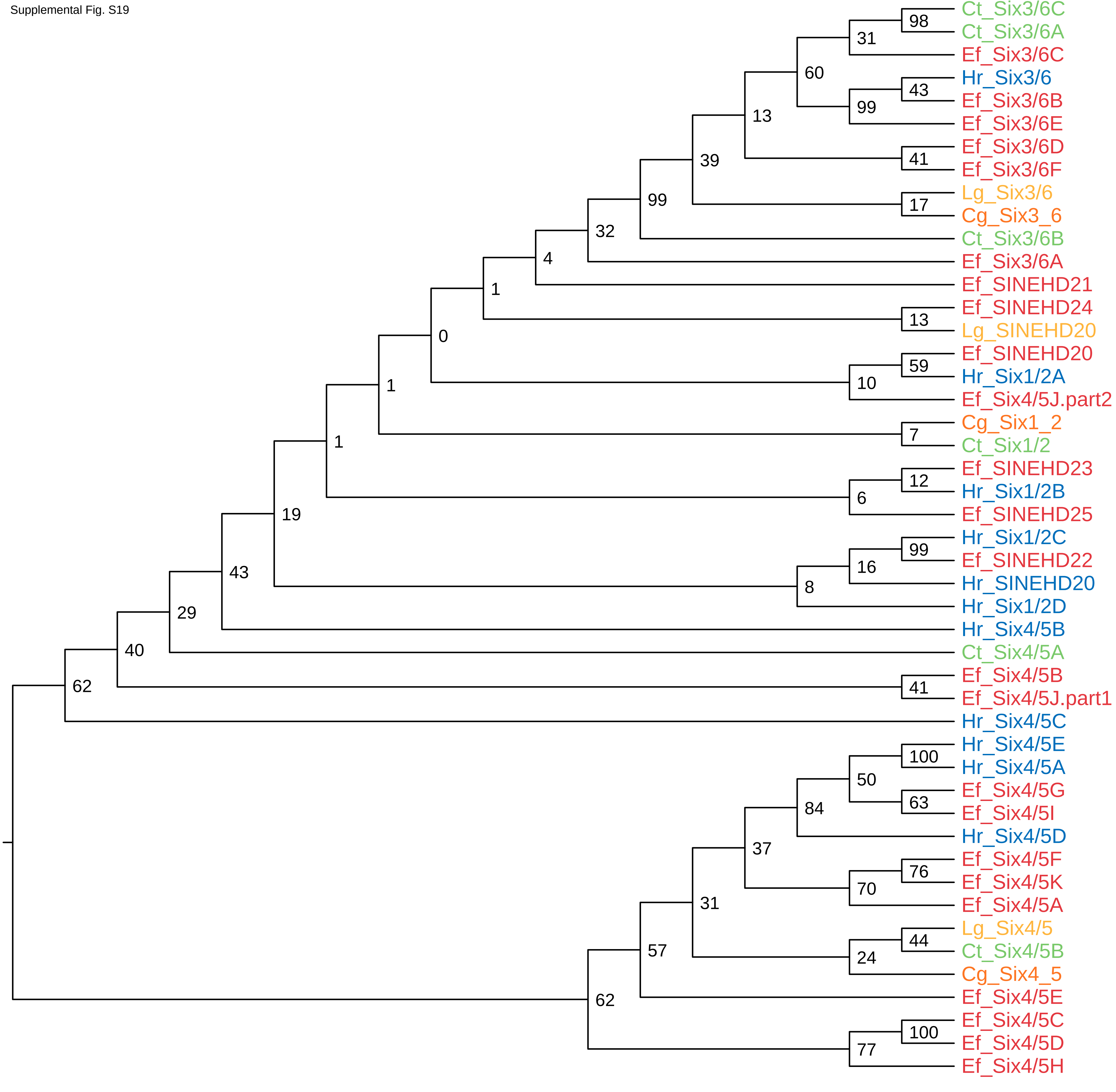

Supplemental Fig. S20

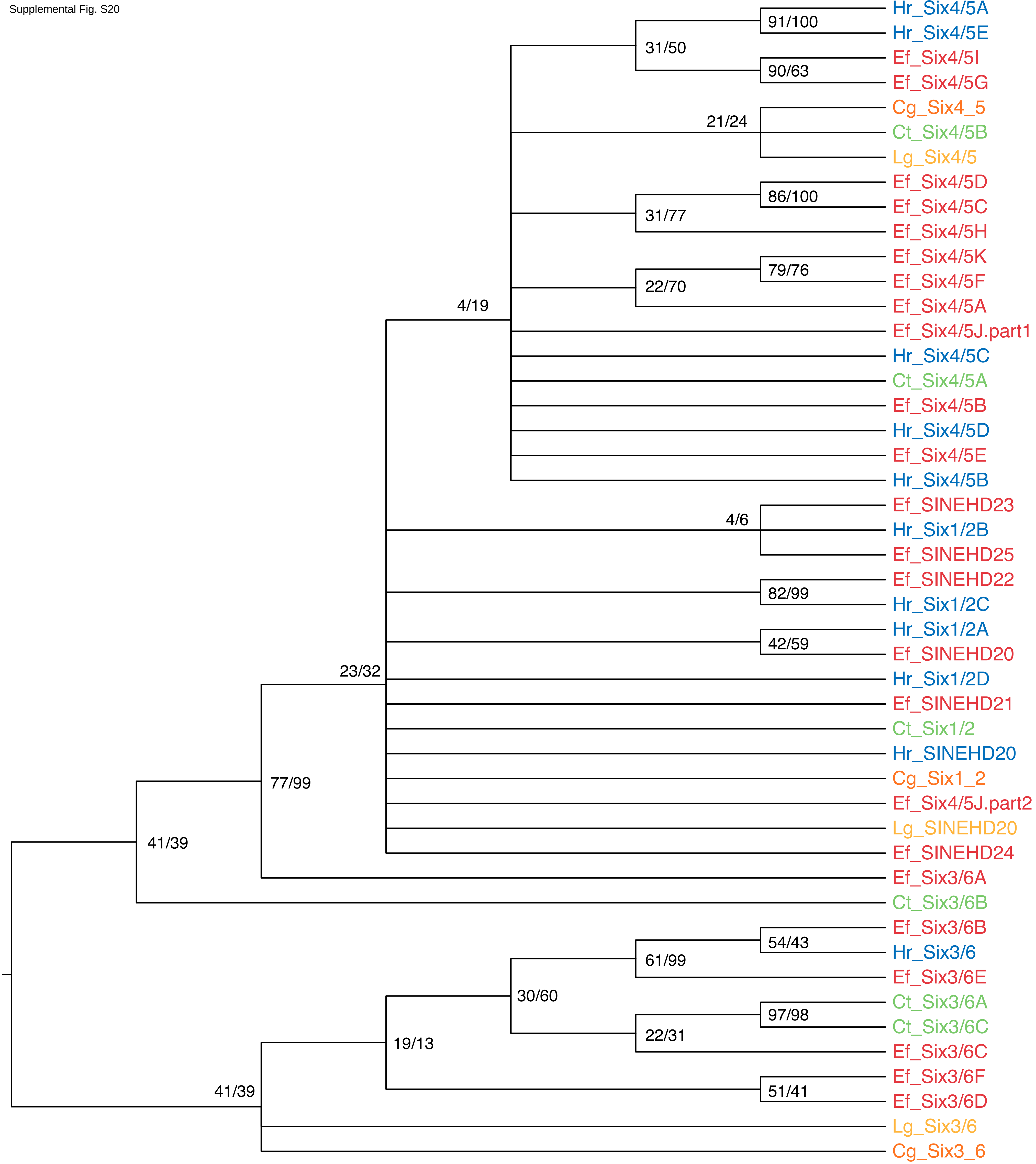

Supplemental Fig. S21

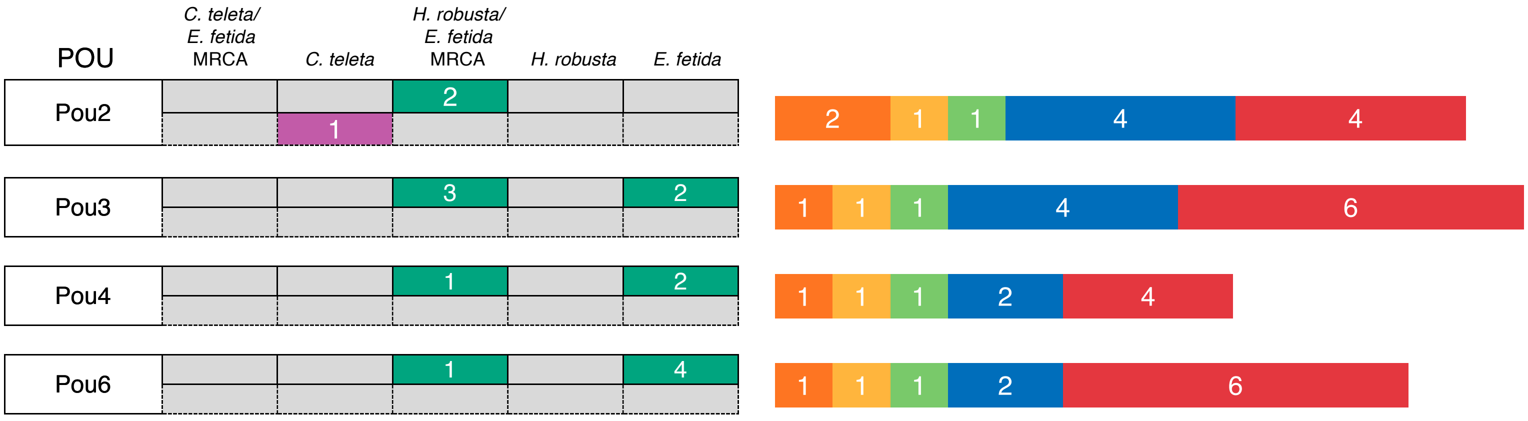

Supplemental Fig. S22

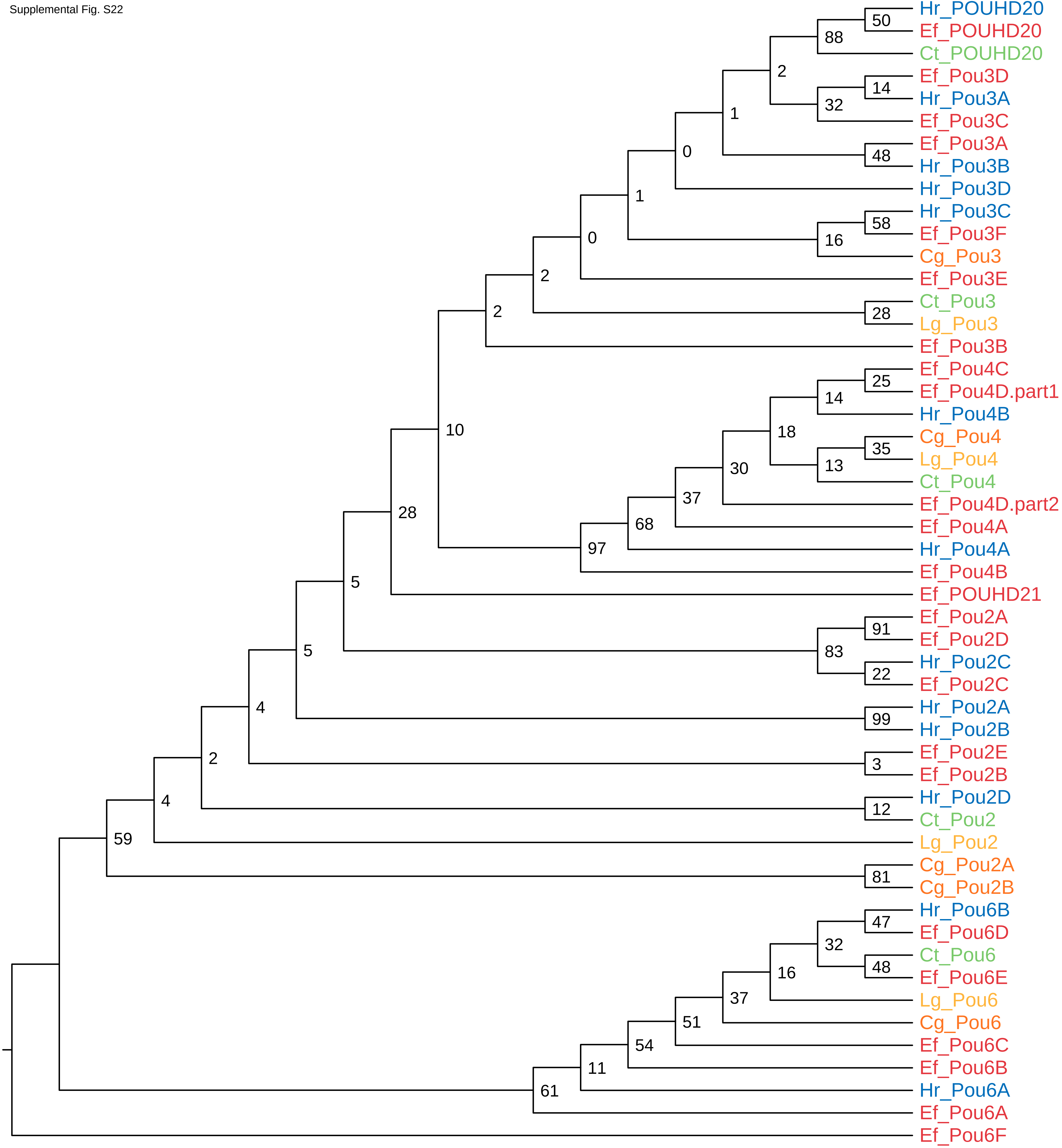

Supplemental Fig. S23

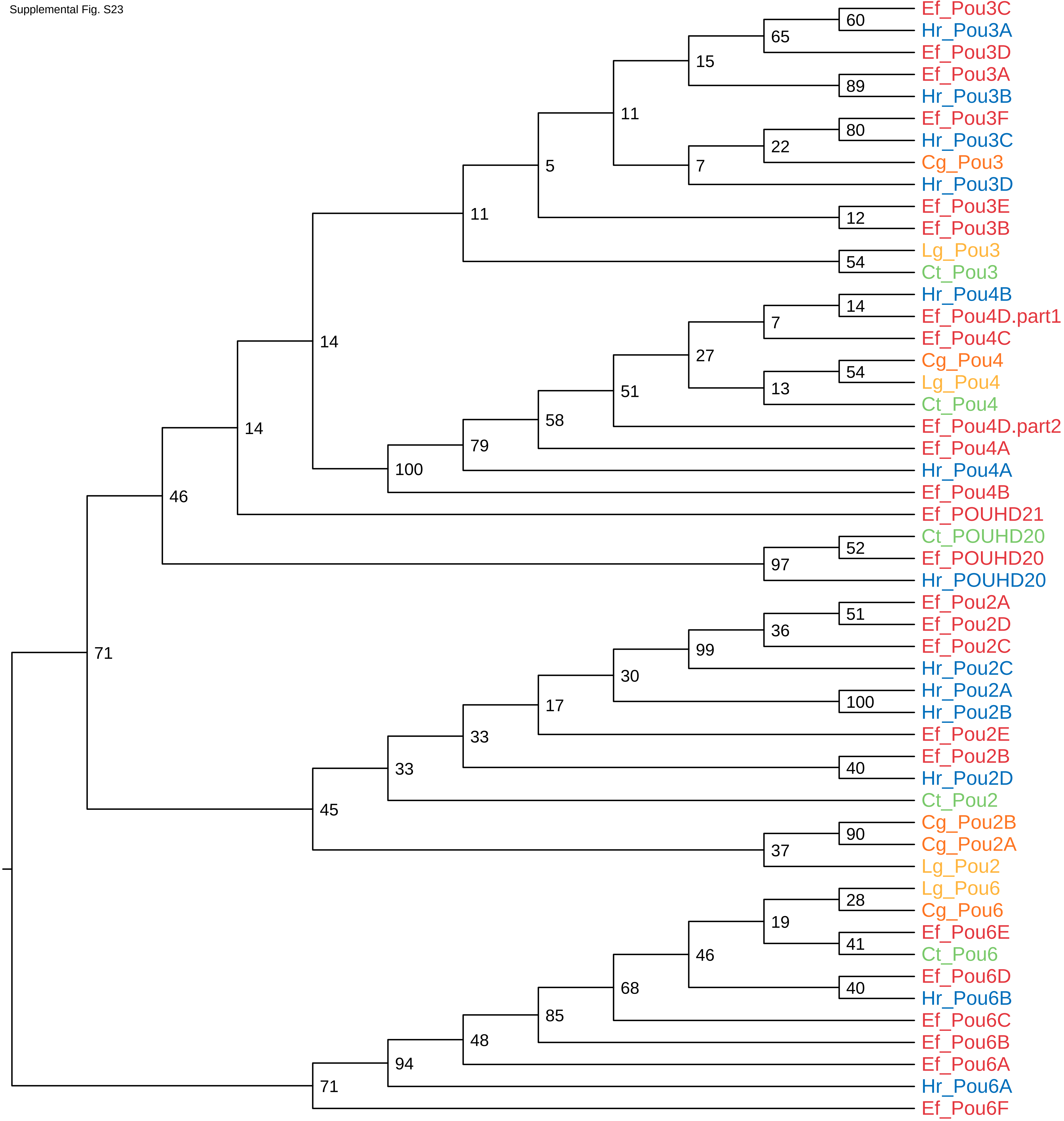

Supplemental Fig. S24

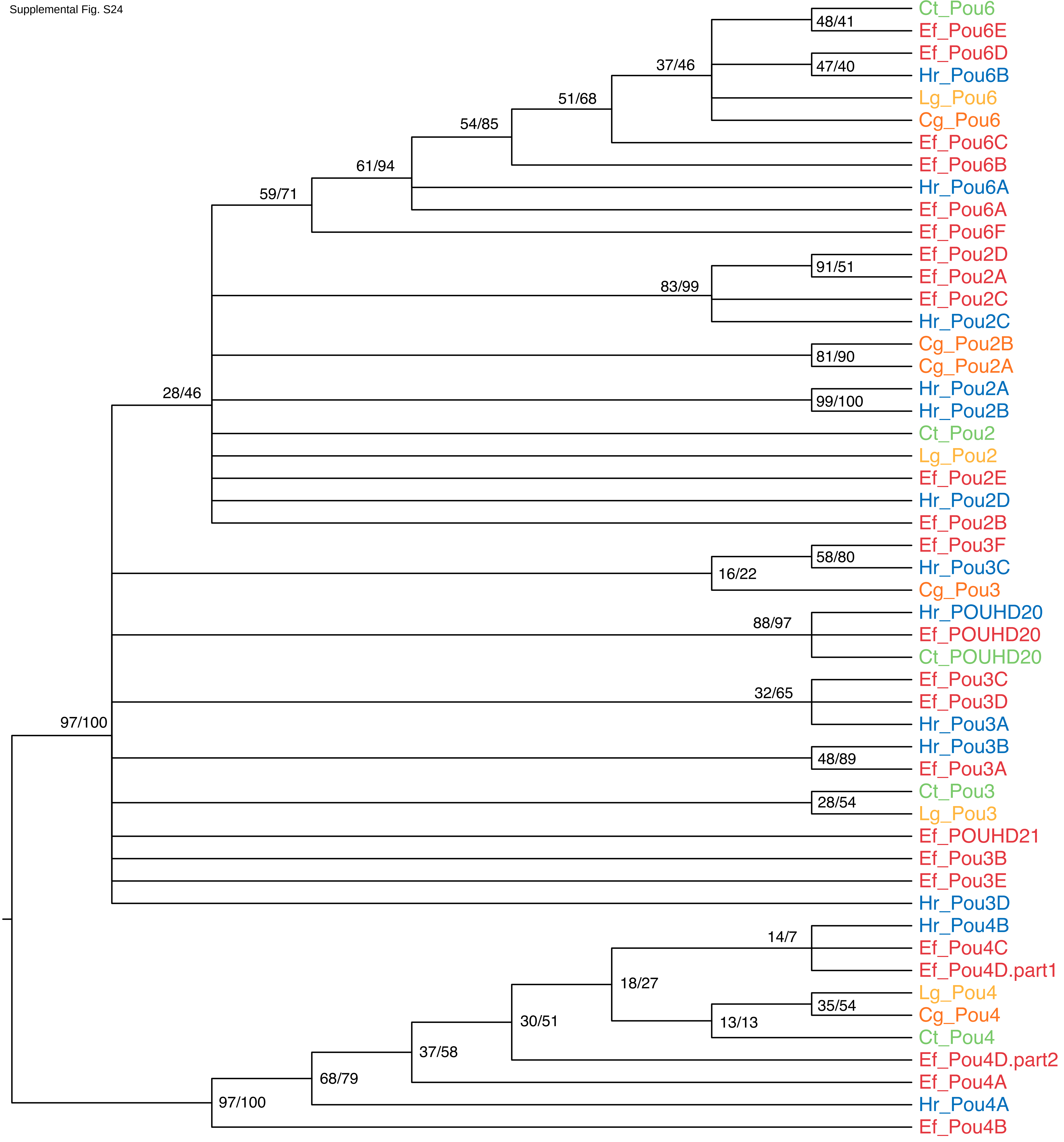

Supplemental Fig. S25

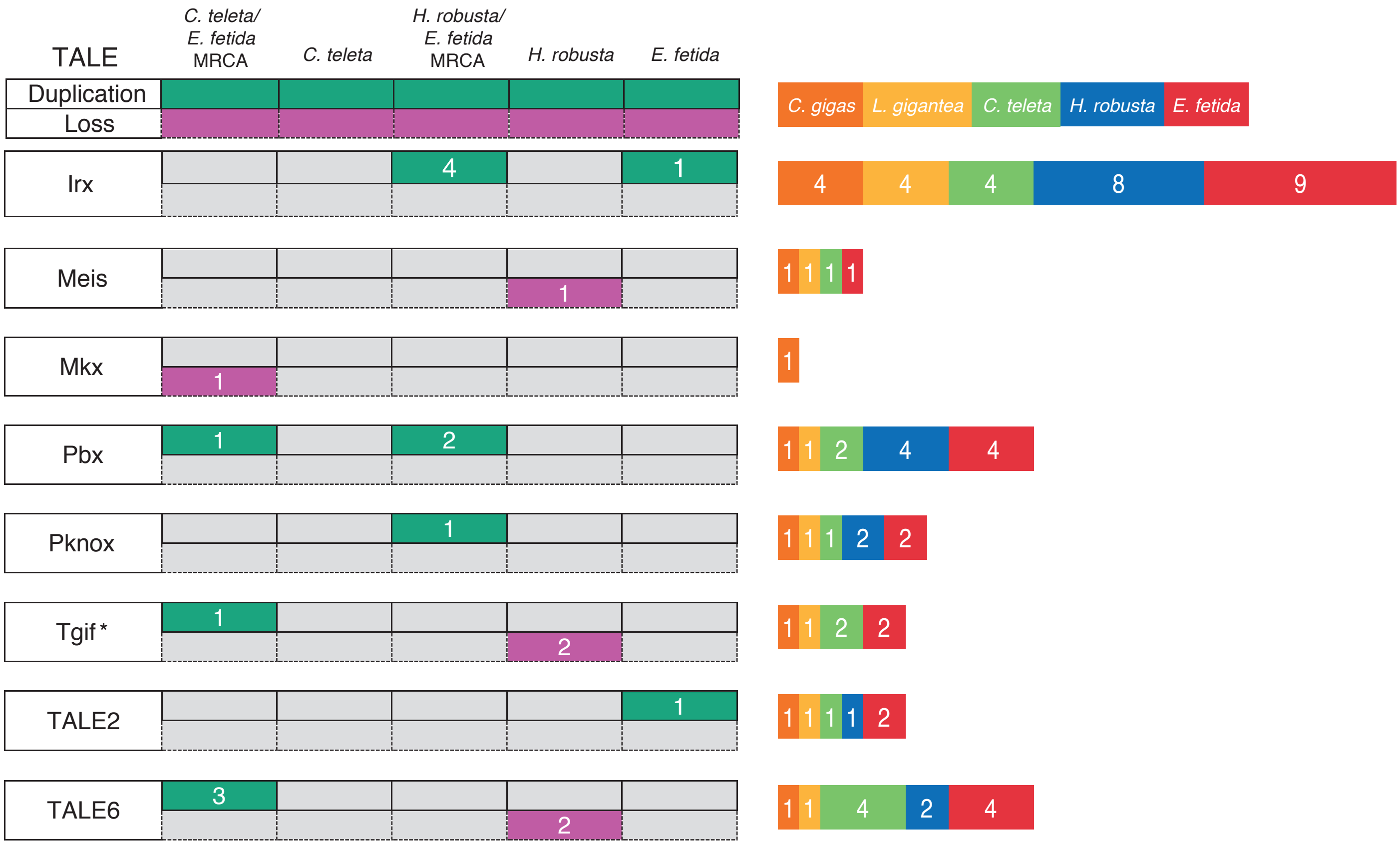

Supplemental Fig. S26

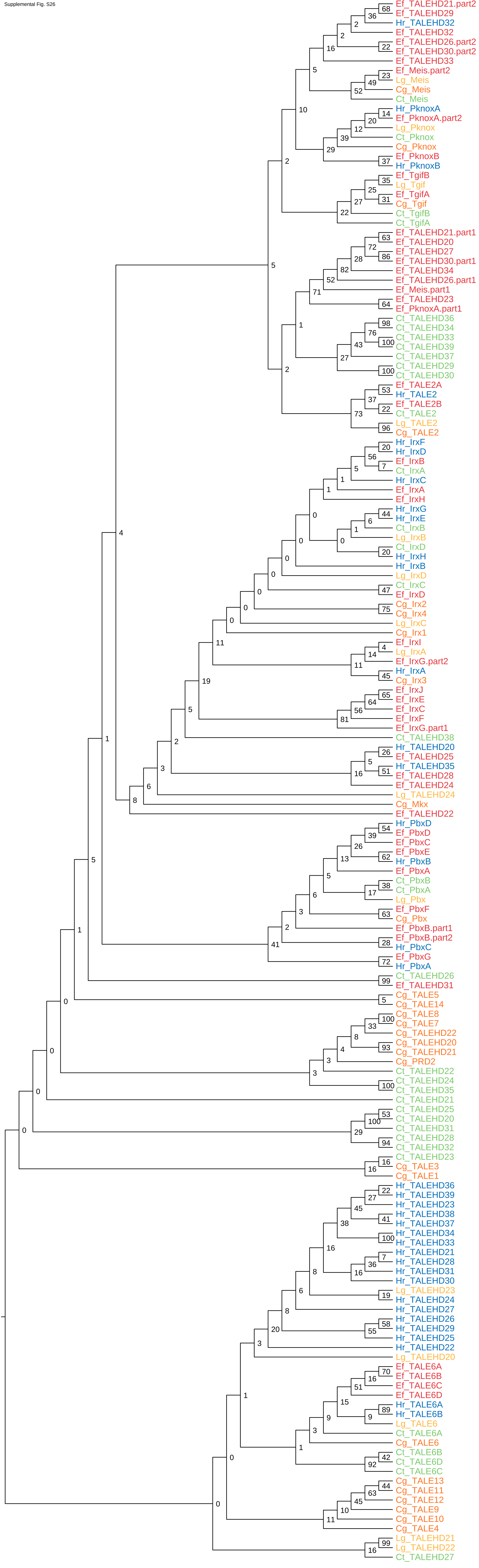

Supplemental Fig. S27

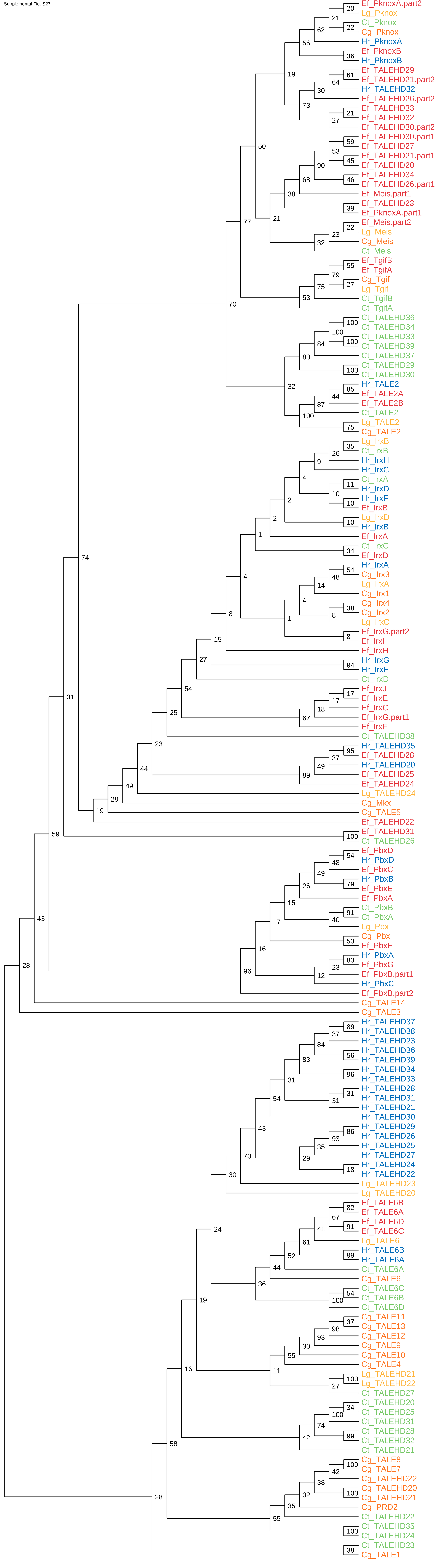

Supplemental Fig. S28

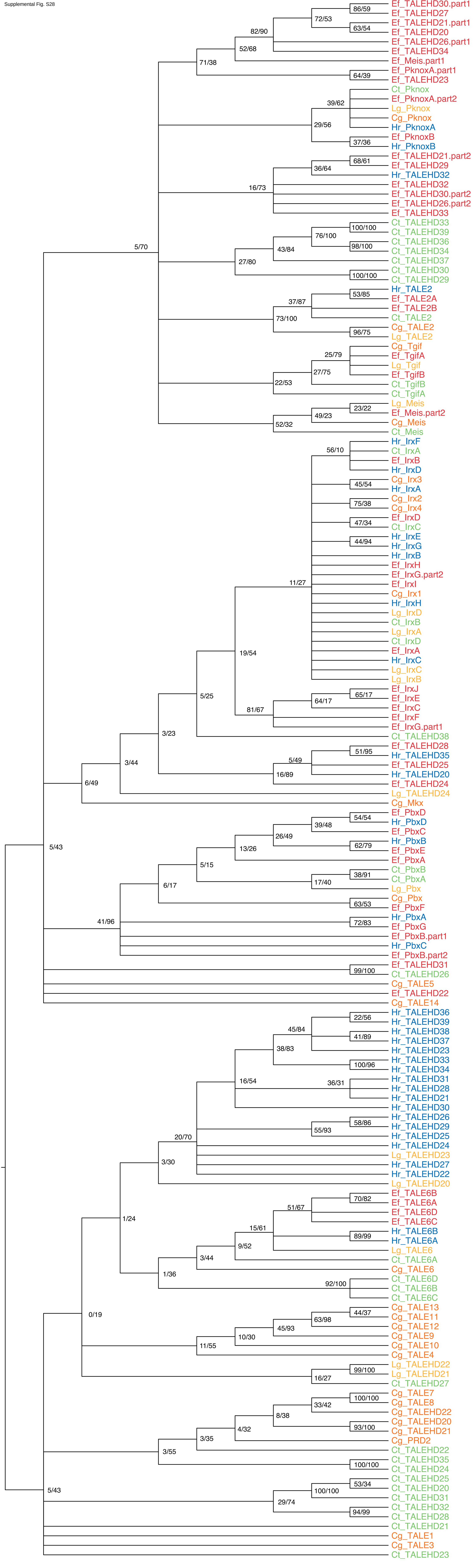

Supplemental Fig. S29

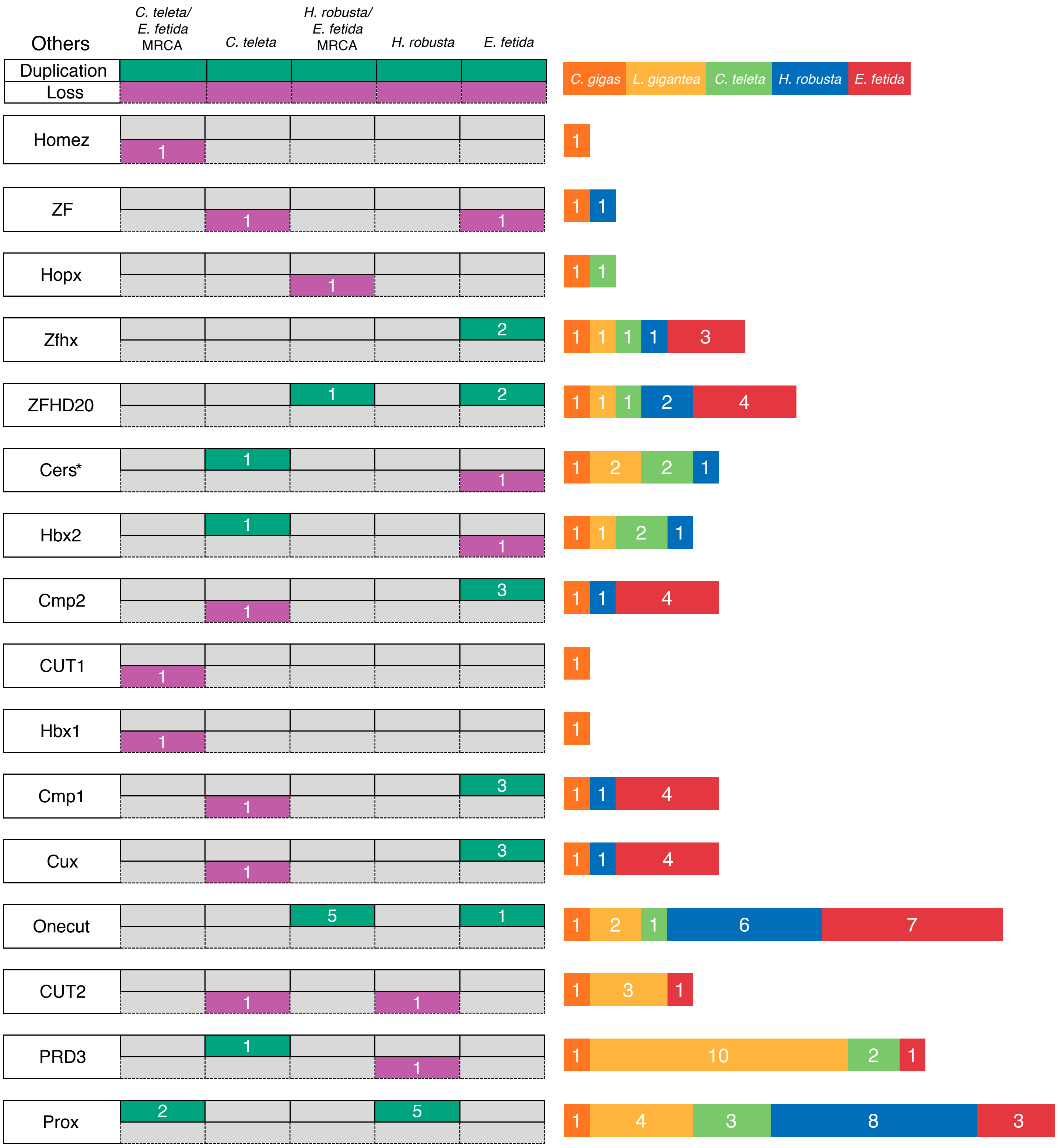

Supplemental Fig. S30

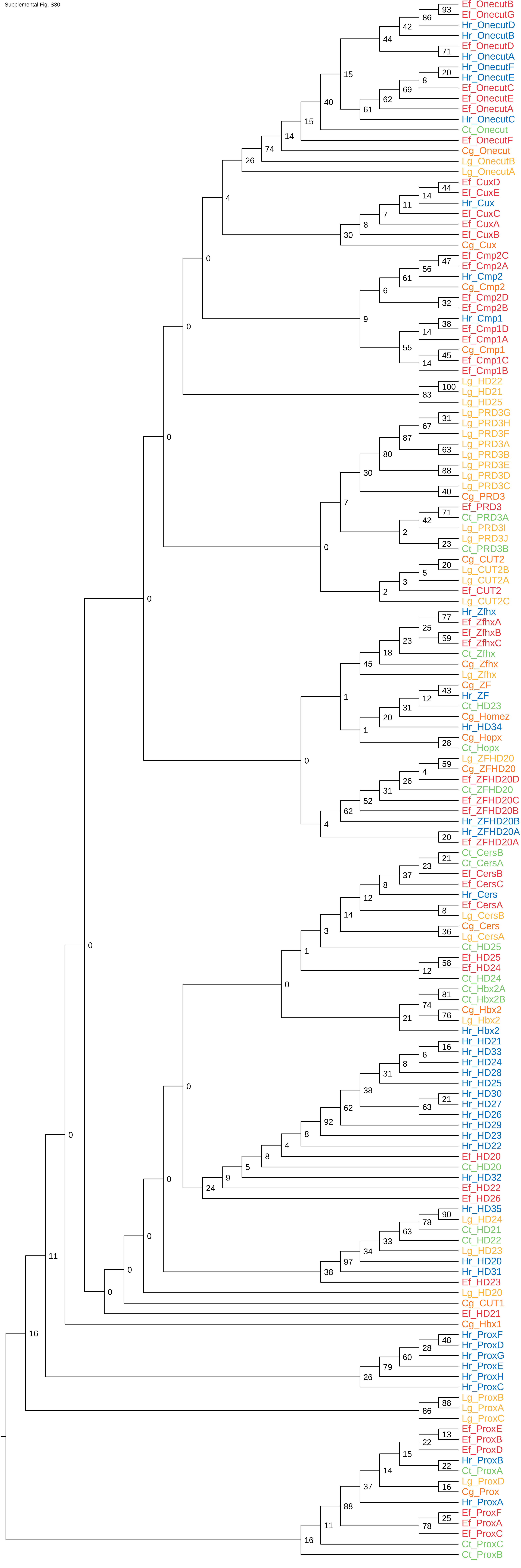

Supplemental Fig. S31

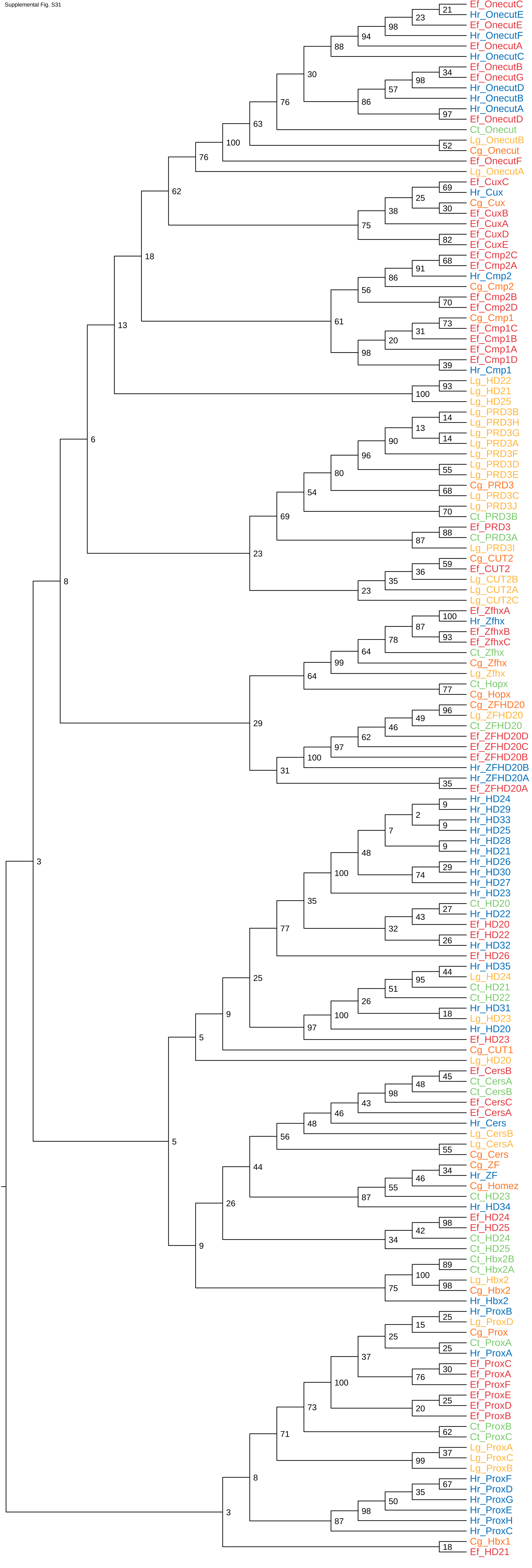

Supplemental Fig. S32

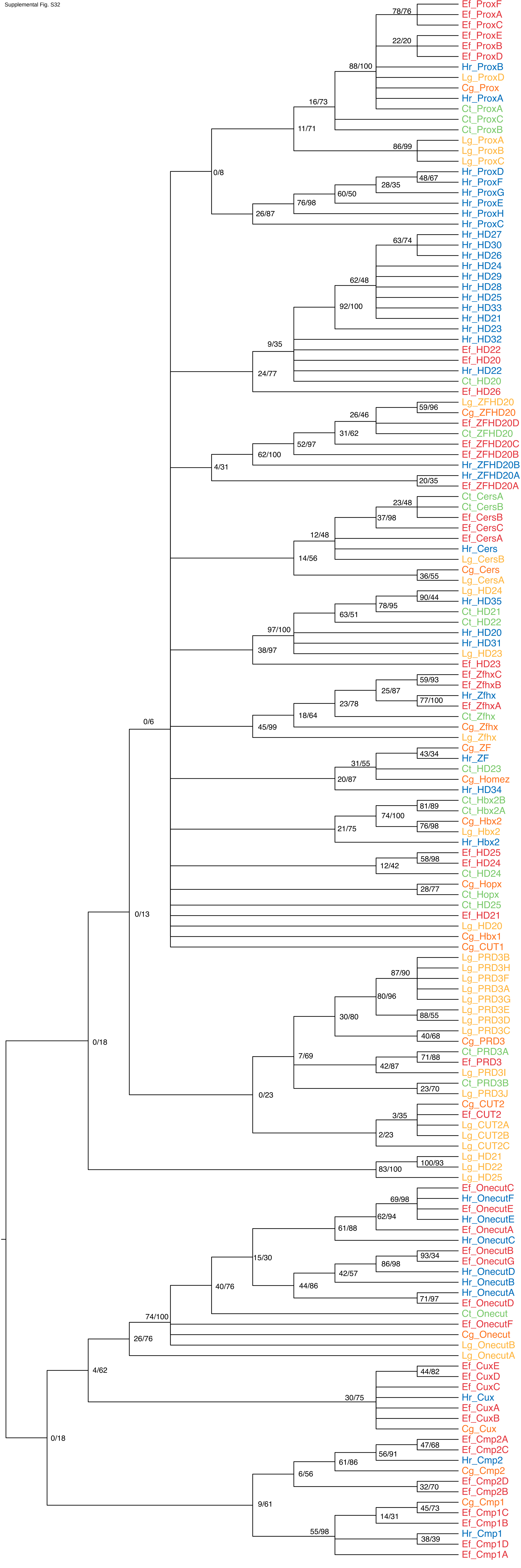

Supplement: Supplementary Data [file supp_evv243_SuppFigs.pdf]
